# Transcriptional regulatory networks of the human gut symbiont *Bacteroides thetaiotaomicron* are uncovered using machine learning

**DOI:** 10.1093/nar/gkaf1166

**Published:** 2025-11-06

**Authors:** Kangsan Kim, Donghui Choe, Sun Chang Kim, Sung Sun Yim, Ki Jun Jeong, Bernhard Palsson, Suhyung Cho, Byung-Kwan Cho

**Affiliations:** Department of Biological Sciences, Korea Advanced Institute of Science and Technology, Daejeon 34141, Republic of Korea; KI for the BioCentury, Korea Advanced Institute of Science and Technology, Daejeon 34141, Republic of Korea; Department of Bioengineering, University of California San Diego, La Jolla, CA 92093, United States; KI for the BioCentury, Korea Advanced Institute of Science and Technology, Daejeon 34141, Republic of Korea; Department of Biological Sciences, Korea Advanced Institute of Science and Technology, Daejeon 34141, Republic of Korea; KI for the BioCentury, Korea Advanced Institute of Science and Technology, Daejeon 34141, Republic of Korea; Graduate School of Engineering Biology, Korea Advanced Institute of Science and Technology, Daejeon 34141, Republic of Korea; Department of Chemical and Biomolecular Engineering, Korea Advanced Institute of Science and Technology, Daejeon 34141, Republic of Korea; Graduate School of Engineering Biology, Korea Advanced Institute of Science and Technology, Daejeon 34141, Republic of Korea; Department of Bioengineering, University of California San Diego, La Jolla, CA 92093, United States; KI for the BioCentury, Korea Advanced Institute of Science and Technology, Daejeon 34141, Republic of Korea; Department of Biological Sciences, Korea Advanced Institute of Science and Technology, Daejeon 34141, Republic of Korea; KI for the BioCentury, Korea Advanced Institute of Science and Technology, Daejeon 34141, Republic of Korea; Graduate School of Engineering Biology, Korea Advanced Institute of Science and Technology, Daejeon 34141, Republic of Korea

## Abstract

*Bacteroides thetaiotaomicron* VPI-5482 is a prominent human gut symbiont of increasing importance to human health and therapeutic applications. Despite its significance, the transcriptional regulatory network (TRN) governing its survival and resilience *in vivo* remains poorly understood. Here, we present BtModulome, a comprehensive transcriptional regulatory framework derived from independent component analysis of 461 RNA-seq datasets spanning diverse niche-specific conditions and genetic backgrounds. This analysis revealed the BtModulome consisting of 110 independently modulated gene sets (iModulons), explaining 72.9% of the variance in the RNA-seq dataset. We validated strong associations with 39 known regulators and identified 311 novel regulator–regulon relationships, accounting for 22.4% expansion of the known TRN of *B. thetaiotaomicron*. In addition, we functionally characterized 11 ECF-σs, including SigW-1, which orchestrates arylsulfatase expression critical for host colonization, and SigH-1, which mediates (p)ppGpp-dependent stringent response. Integration of iModulon activities with multi-omics datasets provided mechanistic insights into stress responses and carbon utilization both *in vitro* and *in vivo*. This comprehensive TRN framework establishes a foundation for understanding adaptive mechanisms in gut commensals and demonstrates the utility of module-centric analysis for biological discovery in non-model organisms.

## Graphical Abstract

**Graphical Abstract:**
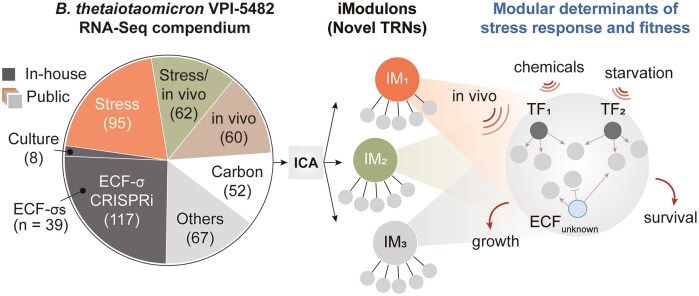

## Introduction

*Bacteroides thetaiotaomicron* represents a model symbiont of the human intestinal microbiota with critical implications for gut homeostasis and human health [1]. Its genetic tractability [2–4] and capacity for long-term intestinal colonization [5] make it a promising candidate for therapeutic applications [6]. The remarkable adaptability of *B. thetaiotaomicron* in the dynamic gut environment is orchestrated by complex transcriptional regulatory networks (TRNs) that coordinate diverse biological processes, including glycan utilization [7], metabolic versatility [8], immune evasion [9], and stress response [10]. In this regard, understanding these regulatory networks is crucial for engineering gut microbiota-based therapeutics and advancing our comprehension of host-microbe interactions.

A distinguishing feature of *B. thetaiotaomicron* is its extensive repertoire of 54 sigma factors, including 50 extracytoplasmic function σ-factors (ECF-σs) that are presumed to coordinate responses to external signals [11]. Nearly half of these ECF-σs are embedded within polysaccharide utilization loci (PULs), suggesting their involvement in nutrient acquisition and competitive fitness [12]. Recent studies have revealed crucial roles for specific ECF-σs, such as ECF sigma factor in oxidative stress, termed EcfO, in oxidative-stress responses in *Bacteroides fragilis* [13], and *Das1* and its cognate anti-σ factor *Dma1* in outer membrane vesicle formation [14]. However, the functions and regulatory networks of many ECF-σs in *B. thetaiotaomicron* remain largely unknown, necessitating a systematic approach to understand their roles in stress response and host-microbe interactions.

Independent component analysis (ICA), a signal decomposition algorithm [15], has emerged as a powerful tool for dissecting regulatory structures in prokaryotic transcriptomes [16]. This approach decomposes gene expression data into independently modulated groups (iModulons), enabling the identification of co-regulated genes and their regulatory relationships [17, 18]. ICA has successfully facilitated the mechanistic understanding of cellular processes [17, 19], functional annotation of hypothetical genes [20, 21], and characterization of adaptive transcriptional responses [22].

Here, we apply ICA to a comprehensive RNA-seq compendium comprising 461 transcriptome profiles across 153 distinct conditions, including diverse stress conditions and genetic perturbations. Our analysis reveals 110 iModulons that collectively explain 72.9% of transcriptome variance, significantly expanding the known TRN of *B. thetaiotaomicron*. Through integration of CRISPRi-mediated repression of 39 ECF-σs with iModulon analysis, we functionally characterize 11 previously uncharacterized ECF-σs. We present detailed characterization of four ECF-σs, including SigR-1, BT0248, SigW-1, and SigH-1, elucidating their regulatory networks and roles in stress response, colonization, and host adaptation. This systems-level analysis provides a comprehensive framework for understanding the regulatory mechanisms underlying the remarkable adaptability of *B. thetaiotaomicron*.

## Materials and methods

### Strains and media used in this study

The wild-type *B. thetaiotaomicron* VPI-5482 (ATCC 29148), obtained from the Korean Collection for Type Cultures, was used throughout the study. All strains used in this study are listed in Supplementary Table S1. *Bacteroides thetaiotaomicron* strains were cultured in brain heart infusion-supplemented broth (BHIS), anaerobic minimal medium [7], amino acid-restricted anaerobic minimal medium, or Columbia Blood Agar (CBA) (MB-C2146; MBcell) supplemented with 5% defibrinated sheep blood (MB-S1876; MBcell). One liter of BHIS broth contains 37 g of BHI powder (237500; BD Bacto), 5 g of yeast extract (212750; BD Bacto), 0.5 g l^−1^ L-cysteine hydrochloride monohydrate (C0517; TCI) dissolved in dH_2_O, 0.2 mM L-histidine (H6034; Sigma–Aldrich)-1.9 µM hemin (51 280; Sigma–Aldrich) solution dissolved in 1 N NaOH (39158–9063; Junsei) adjusted to pH 8, and 1 µg ml^−1^ menadione (M5625; Sigma–Aldrich) dissolved in absolute ethanol (100983; Sigma–Aldrich). L-cysteine hydrochloride monohydrate, L-histidine–hemin solution, and menadione were filter-sterilized using a Minisart® 0.2 µm syringe filter (16534K; Sartorius) and added to the autoclaved media immediately prior to inoculation. Anaerobic minimal medium contains 100 mM of KH_2_PO_4_ (84185–0350; Junsei), 15 mM of NaCl (S7653; Sigma–Aldrich), 8.5 mM of (NH_4_)_2_SO_4_ (A4418; Sigma–Aldrich), 0.5 g l^−1^ L-cysteine hydrochloride monohydrate, 0.2 mM L-histidine-1.9 µM hemin, 50 µM CaCl_2_ (C2661; Sigma–Aldrich), 100 µM MgCl_2_ (M8266; Sigma–Aldrich), 1.4 µl FeSO_4_$\cdot $7H_2_O (215 422; Sigma–Aldrich), 1 µg ml^−1^ menadione, and 5 ng ml^−1^ vitamin B12 (V2876; Sigma–Aldrich). Glucose (G7021; Sigma–Aldrich) was added to the minimal medium at final concentration of 0.5% and filter-sterilized. For amino acid-restricted minimal medium, L-histidine was omitted from the recipe. Cell cultures were routinely performed in a 150 ml serum bottle containing 50 ml of culture media purged with N_2_/CO_2_ (90:10) gas at a pressure of 80 kPa. Antibiotics and inducers, including 25 µg/ml erythromycin (E5389; Sigma–Aldrich), 200 µg/ml gentamicin (G1264; Sigma–Aldrich), and 100 ng/ml anhydrotetracycline (37 919; Sigma–Aldrich), were added where appropriate.

### Molecular cloning

Plasmids and primers used in this study are listed in Supplementary Tables S1 and S2. All clonal vectors used in this study were propagated in PIR1 One Shot *Escherichia coli* (C101010; Invitrogen) or *E. coli* S17-1 λ *pir*. Phusion high-fidelity DNA polymerase (F534S; Thermo Fisher Scientific) and HotStarTaq DNA Polymerase (203443; Qiagen) were used for polymerase chain reaction (PCR) amplification of DNA fragments. All primers were synthesized by Macrogen. Final vector constructs were validated by Sanger sequencing. Isopropyl β-D-1-thiogalactopyranoside (IPTG)-inducible, catalytically inactive Cas9 (dCas9) expression vector was amplified using pMM710 [23]. The purified amplicon was ligated into pLGB13 backbone [24] using In-fusion HD cloning kit (639649; Takara). The dCas9 expression vector contained nearly 2.5 kbp homology arms to induce homologous recombination in the genomic region flanking BT0418, a locus with consistently high expression across different laboratory conditions (Supplementary Table S3). An expression vector containing guide RNA scaffold was amplified using pMM553 as template [23], generating psgRNA_BfP1E6. Single guide RNAs (sgRNA) targeting specific ECF-σs in *B. thetaiotaomicron* were designed using CRISPOR [25]. The sgRNA complementary to ECF-σs, the non-target control, and psgRNA_BfP1E6 backbone were amplified with an overhang sequence containing BpiI Type II restriction enzyme recognition site. DpnI-treated, purified amplicons were digested with BpiI FastDigest enzyme (FD1014; Invitrogen) and were ligated using T4 DNA ligase (EL0011; Invitrogen) following the manufacturer’s protocol. The gene knockout vectors used in this study were cloned using pLGB13 backbone [24] and ∼1 kbp homology arms flanking target genes amplified from the *B. thetaiotaomicron* genome. A total of ∼2 kbp homology arms were ligated using overlap-extension PCR amplification, purified, and ligated with pLGB13 backbone using the In-fusion HD cloning kit.

### Conjugation and selection conditions

The vectors were integrated into the *B. thetaiotaomicron* genome via conjugation using *E. coli* S17-1 λ *pir*, or through tri-parental mating using an *E. coli* strain harboring helper plasmid RK231, as described previously [23]. Briefly, mid- to late-exponential cultures of *B. thetaiotaomicron*, conjugation donor, and conjugation helper strains were harvested and washed twice with BHIS. Washed cell pellets of *B. thetaiotaomicron*, conjugation donor, and helper strains were mixed at 10:1:1 cell mass ratio. Fifty microliters of cell mixtures were spotted onto CBA plates and incubated aerobically at 37°C for 18 to 24 h. Mating spots were resuspended in 3 ml of BHIS liquid medium, streaked onto BHIS plates supplemented with 25 µg/ml erythromycin and 200 µg/ml gentamicin, and incubated anaerobically at 37°C.

### Counterselection conditions

Excision of genome-integrated (pLGB13-based) plasmid backbones via double-crossover events was monitored using anhydrotetracycline-inducible toxin effector *ss*-*bfe1* counter-selection marker. Briefly, PCR-confirmed, erythromycin-resistant *B. thetaiotaomicron* containing plasmid cointegrates were inoculated in non-selective BHIS liquid medium. Cultures were incubated anaerobically at 37°C overnight to allow for second recombination of plasmid cointegrates. Overnight cultures were streaked on BHIS agar supplemented with 100 ng/ml anhydrotetracycline to select for second recombination resolvents.

### dCas9 induction

For inducible dCas9 expression, engineered strains were pre-cultured in 5 ml of BHIS liquid medium and grown anaerobically at 37°C overnight. The overnight cultures were plated in pre-reduced CBA plates supplemented with 5% of defibrinated sheep blood and 25 µg/ml erythromycin. A single viable colony in the overnight CBA culture was resuspended in sterile PBS, spot-plated on CBA supplemented with IPTG (I5502; Sigma–Aldrich) and erythromycin at final concentrations of 100 µM and 25 µg/ml, respectively. Inoculums were incubated anaerobically at 37°C for 12 h before harvest.

### Construction of gene deletion mutants and growth assays

Targeted gene deletion was performed as described previously [24]. The erythromycin-resistant *B. thetaiotaomicron* single clones harboring a gene knockout vector were transferred on non-selective BHIS liquid medium. Overnight cultures were inoculated at an initial OD_600_ = 0.05 in respective culture media. Incubation was performed on a BioTek Epoch microplate reader at 37°C with shaking (206 rpm double orbital) for 24 h, using a default 48-well configuration with a working volume of 300 µl. All plates were sealed using Breathe-Easy® sealing membrane (Z380059; Sigma–Aldrich). The absorbance at A_600_ was measured at 10-min intervals. Growth assays on bile salts were performed using the anaerobic minimal medium supplemented with 0.5% D-glucose (w/v), with or without bile salts (B8756; Sigma–Aldrich) at final concentrations of 0.10% and 0.15% (w/v). Specific growth rates were determined using the Gompertz curve-fitting method, with OD_600_ data from the stationary phase trimmed to ensure good fit (*R*^2^ > 0.98).

### Crystal violet biofilm formation assay

Biofilm formation assay was performed as described previously with minor modifications [26]. Briefly, overnight cultures were inoculated in BHIS and BHIS with bile from bovine and ovine (B8381; Sigma–Aldrich) at final concentrations of 0.25% and 0.50%, respectively. Each inoculum was transferred to polystyrene round-bottom 96-well plates (34096; SPL Life Sciences) in biological duplicates with a working volume of 150 µl. Plates were sealed using a sealing membrane (Z763624; Sigma–Aldrich) prior to incubation. Cell cultures were incubated at 37°C for 48 h. The supernatant was removed from each well by pipetting. Wells were carefully rinsed with sterile PBS. Biofilms were fixed using 150 µl of Bouin’s solution (BOU-OT; Biognost) for 10 min. Wells were washed once with distilled water by immersion and flicking, air-dried, and stained with 150 µl of 1% crystal violet solution (212525; BD) for 10 min. Wells were washed twice with distilled water and air-dried. Stained biofilms were dissolved in 200 µl 1:4 acetone:ethanol mix, and absorbance (OD_575_) was measured using the Synergy H1 microplate reader.

### Reporter expression and fluorescence assay

*Bacteroides thetaiotaomicron* strains expressing the sfGFP were streaked on CBA plates with erythromycin (25 µl/ml) and cultured anaerobically for at least 24 h at 37°C. Colonies were inoculated in 2 ml of BHIS liquid medium with erythromycin (25 µl/ml) and grown anaerobically at 37°C. The overnight BHIS cultures were inoculated in fresh 650 µl BHIS liquid medium with erythromycin (25 µl/ml), in the presence or absence of IPTG (100 µM), in a 96 deep-well 2 ml polyprolene plate (34696; SPL Life Sciences) in biological triplicates. To monitor growth, 150 µl of each inoculum in the deep-well plate was transferred to a 96-well culture plate (3595; Corning) and incubated statically on a BioTek Epoch plate reader for 24 h with A_600_ readings taken at 10-min intervals. To obtain fluorescence readouts, aliquots (150 µl) in the deep-well plate were transferred to a polystyrene round-bottom 96-well plate at specific time intervals, centrifuged at 4000 × *g* for 5 min, and resuspended in PBS twice. After 1 h of oxygen exposure, fluorescence and A_600_ readings were taken on a TECAN Infinite F200 PRO microplate reader with excitation/emission of 465/510 with an optimal gain setting.

### Comparative genomics of PerR regulons across *Bacteroidetes genomes*

Protein-coding sequences of 11 *Bacteroides* type strains—*B. cellulosilyticus* DSM 14838, *B. dorei* DSM 17855, *B. eggerthii* DSM 20697, *B. fragilis* NCTC 9343, *B. ovatus* ATCC 8483, *B. stercoris* ATCC 43183, *B. thetaiotaomicron* VPI-5482, *B. uniformis* ATCC 8492, *B. coprophilus* DSM 18228, *B. plebeius* DSM 17135, and *B. vulgatus* ATCC 8482—were obtained from the UniProt Proteomes database. Phylogenetic tree was constructed using PhyloPhlAn 3.0 [27]. BLASTp hits of PerR iModulon queries with an *E*-value cutoff of 1e-20 and a minimum sequence identity of 30% were considered as orthologs. For BLASTp hits of sigR-1, ecf_classify package was used to validate whether the subject sequence corresponds to “ECF114s7” ECF group [28].

### Total RNA extraction and ribosomal RNA depletion

Total RNA was extracted from the collected cells using the RNASnap™ protocol for Gram-negative bacteria [29]. Briefly, cell pellets were resuspended in 100 μl of RNASnap solution [18 mM ethylenediaminetetraacetic acid (15575-020; Invitrogen), 0.025% sodium dodecyl sulfate (15553-027; Invitrogen), 1% β-mercaptoethanol (M3148; Sigma–Aldrich), and 95% formamide (47670; Invitrogen)]. The mixture was incubated at 95°C for 7 min and centrifuged at 16 000 × *g* for 5 min. The supernatants were purified using an RNA Clean & Concentrator-5 Kit (R1016; Zymo Research), following the manufacturer’s instructions. Ribosomal RNA (rRNA) and genomic DNA contaminants were depleted using the custom rRNA depletion method [30]. Briefly, custom-designed oligonucleotide primers complementary to *B. thetaiotaomicron* VPI-5482 rRNA were hybridized with target rRNA transcripts in the total RNA pool (Supplementary Table S4). Hybridase Thermostable RNase H (H39500; Lucigen) was added to specifically degrade the RNA:DNA duplex (anti-rRNA probe hybridized with rRNA transcript), and 12 U DNase I (M0303L; New England Biolabs) was added to remove genomic DNA fragments and primer dimers.

### Transcriptome sequencing

RNA-seq libraries were constructed with 10–100 ng of rRNA-depleted RNA using a TruSeq Stranded Messenger RNA Library Prep Kit (20020595; Illumina), according to the manufacturer’s protocol. The resulting RNA-seq libraries were quantified using the Qubit double-stranded DNA HS Assay Kit (Q33231; Thermo Fisher Scientific) with a Qubit 2.0 fluorometer and quality-checked using a TapeStation 2200 equipped with a High Sensitivity D1000 Screen Tape (5067-5592; Agilent). The library was sequenced using the HiSeq X platform with 150 cycles of a paired-end reaction.

### Quantitative PCR

Approximately 1 µg of total RNAs extracted from *B. thetaiotaomicron* expressing dCas9 and single-guide RNAs (sgRNAs) cultured on CBA for 12 h were reverse-transcribed into complementary DNA (cDNA) by using SuperScript II reverse transcriptase (18064014; Invitrogen) with random hexamers. Each quantitative reverse transcription (qRT-PCR) reaction mixture contained a 100-fold diluted cDNA, 2 × KAPA SYBR® FAST Quantitative PCR Master Mix Universal (KK4620; Roche), and 0.2 mM of each primer, with a total reaction volume of 10 μl. The qRT-PCR reaction was performed for each sample in three biological replicates, using a StepOnePlus™ Real-Time PCR System (Applied Biosystems, Waltham, MA, USA). The relative expression of each target gene was calculated by normalization to the quantity of 16S rRNA [31]. Primers used for qRT-PCR of target genes—BT1197, BT1728, BT1817—were designed using the NCBI Primer-BLAST.

### Data processing

Public RNA-seq datasets were downloaded using the prefetch command in SRA-toolkit scripts available at https://github.com/ncbi/sra-tools. SRA accessions of public RNA-seq datasets used in this study are available in Supplementary Table S5 and expression dataset in Supplementary Table S6. SRA files were converted to FASTQ files using the fastq-dump (https://github.com/ncbi/sra-tools/wiki/HowTo:-fasterq-dump). RNA-seq data were processed on a CLC Genomics Workbench (CLC Bio, Aarhus, Denmark). Briefly, raw reads were trimmed using a Trim Sequence Tool with 0.05 quality limit. Reads with two or more ambiguous bases were omitted. The resulting reads were mapped on the *B. thetaiotaomicron* VPI-5482 genome and plasmid assemblies (AE015928.1 and AY171301.1, respectively) in a strand-specific manner (backward) with 2 mismatch cost, 3 indel cost, and 0.9 length and similarity fractions. Randomly mapped reads were discarded. The input dataset format for ICA is log-transformed transcript per million (TPM).

### Computing robust components with ICA

ICA was used to decompose the RNA-seq dataset as described previously [32]. The log_10_(TPM + 1) transformed values were batch-normalized to reference conditions specified in each experiment. Next, the scikit-learn implementation [33] of the FastICA [34] was iterated 100 times with random seeds and a convergence tolerance of 10^−7^ to obtain robust independent components (ICs). The resulting ICs were clustered using the scikit-learn implementation of the DBSCAN [35] to identify robust ICs, using an epsilon of 0.1 and a minimum cluster seed size of 50. The distance metric was computed using the absolute value of the Pearson correlation ($\rho $) between components *x* and *y* using Equation (1)


(1) \begin{eqnarray*}
{{d}_{x,y}} = 1 - \|{{\rho }_{x,y}}\|
\end{eqnarray*}

To determine the optimal dimension, we applied above procedure to the dataset multiple times for dimensions between 20 and 300 with a step size of 20. The optimal dimensionality of 280 was chosen, where the number of non-single ICs was equal to the number of final components. This generated M (gene weight) and A (activities) matrices decomposed from the gene expression profile for 110 robust ICs (Supplementary Tables S7 and S8).

The iModulon activity matrix A represents the condition-dependent activity of each IC across all RNA-seq samples. It was computed as part of the ICA decomposition of the expression matrix *M* = *X* × *A*, where *X* is the original gene expression matrix (Supplementary Table S6). The activity of a given iModulon in each condition thus reflects the regulatory signal inferred from gene co-expression patterns and can be interpreted as a proxy for the underlying transcriptional regulator’s activity [16].

Gene membership within each IC was defined based on gene weights in the corresponding component vector M. The significance of gene weights in each IC was determined by the D’Agostino *K^2^* test statistic of the scikit-learn [36]. This test identifies a threshold beyond which gene weights significantly differ from background noise, thereby identifying the subset of genes most strongly contributing to the iModulon. The optimized threshold was determined using the “optimize_cutoff = True” setting in the Pymodulon package (https://github.com/SBRG/pymodulon), which maximizes signal detection while minimizing false positives. Genes with absolute weights exceeding this threshold were classified as iModulon members.

### Characterization of iModulons

The enrichment analysis of regulators in each iModulon was performed as described previously [17], using the Python-based gene annotation pipeline in the Pymodulon package. Strain-specific gene annotations were obtained from BioCyc [37] and UniProt [38]. Clusters of Orthologous Groups (COGs) information were generated using the EggNOG mapper [39]. Gene ontology (GO) information was obtained from AmiGO2 [40]. The existing TRN was collected from RegPrecise [41] and published literatures (Supplementary Table S9). Enrichment analysis of GO and KEGG annotations was performed using Fisher’s Exact Test with a False Discovery Rate (FDR) of <10^−2^. iModulons with statistically significant overlaps with known TRNs (FDRs < 10^−4^) were classified as “regulatory” iModulons. All iModulons were manually assigned into eight different user-defined categories—PULs, Uncharacterized, ECF-σ, Metabolism, Structural components, Functional, Stress, and Single gene—based on the shared functions of their gene membership. ECF-σ iModulons contain ECF-σ as the sole or the highly-weighed TF but with ambiguous functional attributes. The naming convention for *B. thetaiotaomicron* ECF-σs described in this study is based on the ECF Hub annotations [28].

### Statistical analysis

All statistical analyses were performed using the scipy.stats module in Python. Unless otherwise noted, bar graphs represent the means ± SD. For comparing statistical differences between means of multiple samples, a two-sample independent *t*-test was used. Statistical tests used and *P* values are noted in figures and figure legends. Correlation analysis was performed using a Pearson correlation, using the scipy.cluster and scipy.spatial module in Python.

## Results

### ICA reveals 110 independently modulated groups of genes in *B. thetaiotaomicron*

ICA enables decomposition of complex transcriptome data into independently modulated gene groups, termed iModulons [17]. These regulatory modules function analogously to regulons, comprising functionally related genes that show coordinated expression patterns across diverse conditions. Such organizational structures provide insights into the collective activity and regulation of their constituent genes under varying environmental and genetic contexts [17].

**Figure 1.**
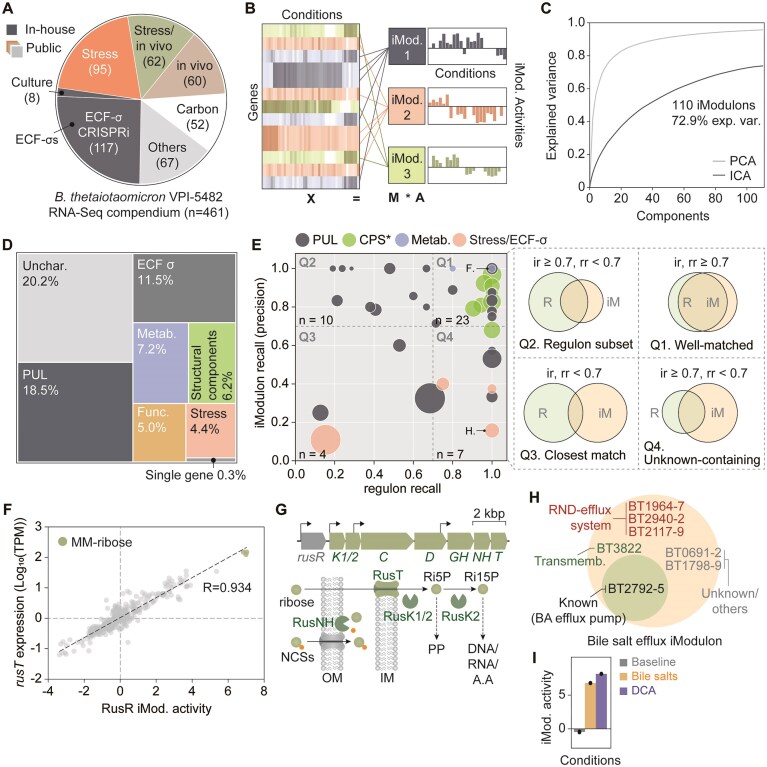
ICA-based decomposition of transcriptional regulatory signals in *B. thetaiotaomicron* from a compendium of transcriptomic datasets. (**A**) The composition of *B. thetaiotaomicron* VPI-5482 RNA-seq data collected across different environmental backgrounds. A total of 461 RNA-seq data sets are represented. (**B**) A schematic representation of signal decomposition. The log-transformed gene expression data, denoted as *X*, is decomposed into co-regulated iModulons, denoted arbitrarily as iMod. 1, 2, and 3, and the activity of the iModulons across conditions, shown as a histogram for the entries in the corresponding row of the activity matrix A. (**C**) ICA analysis generated 110 iModulons with 72.9% of explained variance in the dataset. Since the iModulons are knowledge-enriched, this corresponds to fundamental explanation. In contrast, the classical explanation by PCA is a statistical measure. (**D**) A treemap of the combined explained variances of iModulons grouped by their category of cell functions they belong to. PUL, polysaccharide utilization loci. ECF σ, extracytoplasmic function sigma factor. (**E**) Scatter plot of iModulon recall (or precision) and regulon recall of the significantly enriched 44 (out of 110) regulatory iModulons. Each circle represents individual iModulons. Circle size is in proportion to the number of genes in each iModulon. Colors represent iModulon functional categories, same as in panel (D). Two specific iModulons labeled as F and H denote iModulons in panels (F–I). The accuracy of iModulon predictions can be evaluated using iModulon recall and regulon recall metrics, where iModulon recall is the fraction of known regulons in an iModulon, and regulon recall is the fraction of iModulons that are in match within a known TRN. Based on the two measures, iModulons can be classified into four groups—“well-matched,” “regulon subset,” “unknown-containing,” and “closest match.” The four Venn diagrams correspond to the four quadrants of the plane. CPS: capsular polysaccharide biosynthesis belongs to “Structural components” category, ir: iModulon recall, rr: regulon recall. (**F**) A strong positive correlation was observed between RusR iModulon activity with the gene expression of putative ribose transporter, *rusT*. Notably, the highest RusR iModulon activity and *rusT* expression are both observed in minimal medium supplemented with ribose across the 461 RNA-seq experiments in the compendium. (**G**) RusR iModulon is composed of the Rus PUL (BT2803–2809) required for ribose utilization, as per the literature evidence. The proposed role of RusT involves transport of free ribose into the cytoplasm. Ri5P: D-ribose-5-P, Ri15P: D-ribose-1,5-BP, NCS: nucleosides. (**H**) Members of the Bile salt efflux iModulon contain previously known efflux pumps associated with bile acid tolerance (BT2492–BT2495), as well as newly predicted genes annotated as multidrug resistance/efflux pumps and transmembrane (transporter) proteins. (**I**) Activity of Bile salt efflux iModulon in culture condition datasets (PRJNA983800). DCA: deoxycholic acid.

To dissect the transcriptional architecture of *B. thetaiotaomicron*, we compiled and analyzed 461 RNA-seq datasets from two in-house and twenty public projects (Supplementary Table S5). The dataset encompasses diverse transcriptional responses, with nearly half (46.7%) representing responses to various stress conditions both *in vitro* and *in vivo*, followed by *in vivo* growth/adaptation (17.9%) and carbon source utilization (15.4%) (Fig. 1A and Supplementary Table S5). The compendium includes transcriptional profiles from mutants lacking key regulators of carbohydrate utilization [8], (p)ppGpp-associated genes [10, 42], capsular polysaccharide gene clusters [9], and vesiculation-associated ECF-σ factors [14]. We further expanded this compendium by generating CRISPRi-mediated repression strains targeting 39 of the 50 ECF-σ factors encoded by *B. thetaiotaomicron*, many of which have unknown functions [11] ( Supplementary Fig. S1 and S2; refer to Supplementary Notes S1–S3 more details on RNA-seq data preparation). The resulting dataset captures a broad spectrum of transcriptional responses to both environmental and genetic perturbations.

ICA decomposition of this comprehensive dataset yielded 110 iModulons, collectively termed the BtModulome, which explain 72.9% of total variance in gene expression (Fig. 1B and C). This number and the associated explained variance are comparable to previously reported ICA decompositions in iModulonDB, supporting the robustness of the inferred regulatory structure [16]. These iModulons capture functionally coherent modules, many of which are enriched for genes involved in distinct cellular processes (Fig. 1D). Notably, iModulons enriched for genes of unknown function accounted for 20.2% of the total variance, suggesting novel regulatory relationships. In particular, PULs-associated iModulons represented 18.5% of the total variance, consistent with PULs occupying approximately one-fifth of the *B. thetaiotaomicron* genome [11]. Additionally, ECF-σ-associated iModulons without clear functional classifications captured 11.5% of the explained variance, with eight previously uncharacterized ECF-σ factors contributing to distinct regulatory modules. Unlike PCA, which ranks components solely by the magnitude of variance captured, ICA identifies statistically ICs that often correspond to biologically coherent transcriptional modules [17]. Thus, the variance explained by ICA is an indicator of how much of the transcriptome is governed by discrete, biologically interpretable regulatory signals.

To validate our approach, we evaluated 44 “regulatory iModulons” showing statistical enrichment with known or predicted TRN features (Supplementary Table S10, see “Materials and methods” section). Using iModulon recall (or precision) and regulon recall measurements [17], we classified these iModulons into four distinct groups (Fig. 1E) [19, 22]. The “well-matched” group (iModulon recall and regulon recall $\ge $ 0.7) comprised 23 iModulons, including 7 of 8 capsular polysaccharide synthesis (CPS)-associated iModulons and 14 PUL-associated iModulons, demonstrating effective recapitulation of established regulon structures. The high iModulon recall-regulon recall metrics of CPS-associated iModulons likely reflect their organized operon structure, with cognate transcription factors (TFs) positioned at the 5′ end of each operon (Supplementary Fig. S3) [11].

The ability of iModulons to capture co-regulated expression patterns is exemplified by the RusR iModulon, which regulates ribose scavenging in *B. thetaiotaomicron* [43]. This module fully captured both genetic requirements and condition-specific activation of the ribose utilization operon (Fig. 1F and G). Genes were assigned to each iModulon based on their weight values in the corresponding IC, where only those with large, outlier-like contributions were selected to distinguish meaningful regulatory signals from background noise (Supplementary Fig. S4A and B). The absence of RusR within the iModulon is consistent with the previous report where the expression of *rusR* remained unaltered in the presence of ribose (Supplementary Fig. S4B) [43], suggesting the complexity of TRN [44]. These findings validate our ICA-based approach for reconstructing known TRNs while revealing potentially novel regulatory associations absent from existing networks [17].

### iModulon provides inference on novel regulatory and functional associations

iModulons provide insights into novel regulatory and functional associations, revealing regulatory interactions and stimulons that share common regulatory stimuli [19]. This complexity results in variable iModulon recall–regulon recall metrics across iModulons. The “regulon subset” group (*n* = 10), containing subsets of known TRNs, exhibits high iModulon recall but low regulon recall (Fig. 1E). This pattern typically emerges in large TRNs comprising multiple iModulons, as iModulons are derived from expression patterns rather than direct TF binding [17, 19]. Such subset iModulons likely reflect varying regulatory strengths within TRNs or indicate undiscovered regulatory layers [22] (see Supplementary Note S4 and Supplementary Fig. S5).

iModulons enable functional prediction of uncharacterized genes by capturing co-regulated genes in specific metabolic processes [17, 19]. We identified seven iModulons in the “unknown-containing” group, characterized by low iModulon recall and high regulon recall, suggesting novel regulator–regulon relationships or co-activation patterns. A notable example is the Bile salt efflux iModulon, comprising three operons encoding resistance-nodulation-division efflux pumps, hypothetical proteins, and a transmembrane protein (Fig. 1H). Its specific activation in response to bile salts indicates a role in bile acid resistance (Fig. 1I). Within this group, the BT2792–2795 operon, previously identified as a tripartite multidrug efflux system [45], plays a critical role in bile salt stress tolerance. Further analysis revealed potential roles of putative membrane proteins in bile salt-induced biofilm formation (see Supplementary Note S5 and Supplementary Fig. S6).

Additionally, we identified four “closest match” iModulons showing limited overlap with known regulons, possibly reflecting condition-dependent TF binding strengths or incomplete TRN knowledge [19, 22]. In particular, two multi-polysaccharide utilization iModulons and the NanR iModulon, each encompassing multiple PULs along with carbohydrate-active enzymes, catalytic enzymes, and uncharacterized proteins (Supplementary Table S11), displayed significant regulator enrichment and interpretable activity profiles under various conditions, suggesting potential targets for novel discoveries (see Supplementary Note S6 and Supplementary Fig. S7).

This iModulon-based approach successfully integrates diverse, independent data into a coherent framework that both validates known TRNs and reveals novel regulatory relationships, generating testable hypotheses for future investigation.

### ICA elucidates transcriptional regulatory associations involving ECF-σs

**Figure 2.**
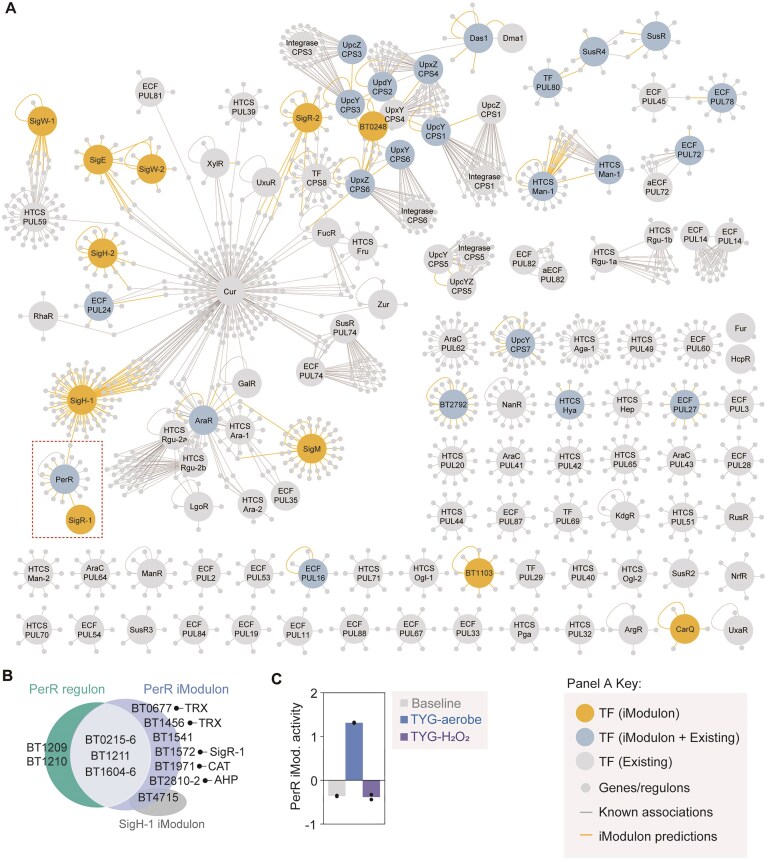
The expanded TRN of *B. thetaiotaomicron*. (**A**) The reconstruction of *B. thetaiotaomicron* TRN expanded using the 39 regulatory iModulons that belong to well-matched, regulon-subset, and unknown-containing categories. Larger circles (hubs) with annotations represent TFs (including sigma factors and TFs), while smaller gray nodes surrounding each hub denote genes with regulatory associations. TRNs within the red dashed box are discussed further in panel (B) and (C) and in Supplementary Fig. S8. (**B**) Predicted regulatory network in PerR. iModulon predicted 10 additional regulatory interactions that involves co-regulation by multiple regulators, including SigR-1 and SigH-1. The extended memberships of the PerR iModulon include thioredoxins (TRX), a catalase (CAT), alkyl hydroperoxide reductases, a transmembrane protein (BT1541), an aminotransferase (BT3935), non-specific DNA-binding protein (BT4715), and SigR-1 (BT1572). (**C**) Condition-specific activation of PerR iModulon in an array of stress-inducing conditions outlined in PRJNA983800 (Supplementary Table S5). Note the sharp increase in PerR iModulon activity in response to aerobic exposure.

To explore novel regulatory networks in *B. thetaiotaomicron*, we reconstructed an expanded TRN based on iModulons analysis. By integrating regulatory–regulon associations from both regulatory iModulons and 11 ECF-σ iModulons with proposed ECF-σ regulation but unclear functional attributes (Fig. 2A and Supplementary Table S10), we identified 1697 regulator–regulon associations, including 311 novel predictions. These 1697 associations represent a 22.4% expansion of the known TRN of *B. thetaiotaomicron* (Supplementary Table S9) and encompass 34 TFs, including 11 previously uncharacterized ECF-σs (Fig. 2A).

A key finding emerged from analysis of the PerR iModulon, which revealed nine additional regulatory interactions involving multiple regulators, including SigR-1 and SigH-1, alongside known RegPrecise-predicted regulons [41] (Fig. 2B and Supplementary Fig. S8A). PerR, a conserved bacterial TF, governs responses to hydrogen peroxide and oxidative stresses [46, 47]. In *B. thetaiotaomicron*, its known regulons include auto-regulatory PerR (BT0215), rubrerythrin (BT0216), cytochrome peroxidase-associated proteins (BT1604-BT1606), and a cytochrome D oxidase-associated operon (BT1209–BT1211). The expanded PerR iModulon network incorporated additional stress response components including thioredoxin proteins, catalase, alkyl hydroperoxide reductases, and metallophosphoesterase (Fig. 2B), aligning with aerobic stress response genes identified in *B. fragilis* [48].

Notably, the network included SigR-1, an ECF-σ known to modulate expression of thioredoxin systems in response to oxidative stresses [49]. While PerR and SigR-1 expression showed strong positive correlation (Pearson *R* = 0.65) across the RNA-seq compendium (Supplementary Fig. S8B), CRISPRi-mediated SigR-1 repression did not lead to changes in PerR regulation compared to the non-target control, suggesting that PerR is regulated independent of SigR-1. DEG analysis, however, suggested potential regulatory associations with other iModulon members, including a thioredoxin family protein (BT0677), cytochrome peroxidase-associated proteins (BT1605–1606), and a catalase (BT1971) (Supplementary Fig. S8C and D). Functional validation showed increased PerR iModulon activity specifically under aerobic exposure, but not H_2_O_2_ treatment (Fig. 2C), possibly reflecting insufficient H_2_O_2_ stress induction in the original study [50] rather than biological significance. These findings highlight iModulon’s capability to identify previously unrecognized TRNs from complex gene expression patterns, enabling discovery of novel regulatory associations and their biological functions.

Next, to assess conservation of PerR iModulon membership, we performed BLASTp searches against ten *Bacteroides* type strains predicted to harbor the PerR regulatory network by the RegPrecise database (Supplementary Fig. 8E and F) [41]. The result revealed a set of PerR iModulon members conserved across species, including PerR, rubrerythrin, the cytochrome D oxidase operon, and an alkyl hydroperoxide reductase subunit (BT2811), which together constitute the core PerR regulon (Supplementary Fig. 8F). Among the SigR-1 orthologous, only those of *B. fragilis* and *B. ovatus*, the phylogenetically close relatives, belonged to the same ECF-σ subgroup “ECF114s7” [28]. In addition, *B. fragilis* and *B. ovatus* exhibited the highest protein similarity and overall conservation (Supplementary Fig. 8F), suggesting that the PerR regulatory architecture, and perhaps the regulation by SigR-1, is conserved in these species. Together, these results suggest that PerR‐dependent regulatory networks are broadly maintained across the genus but have undergone lineage‐specific changes with yet unknown implications.

### Functional characterization of novel ECF-σ factors through ICA-based inference

**Figure 3.**
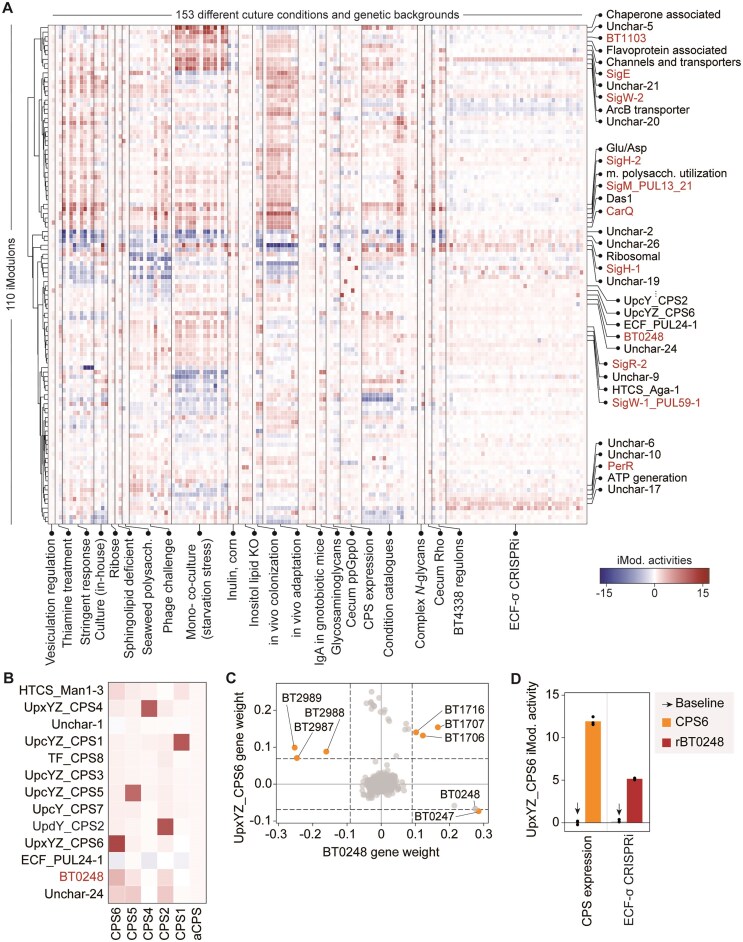
The overview of iModulon activities. (**A**) Hierarchical clustering (distance: Euclidean, method: Ward) of the rows of the Matrix A. Clusters represent multiple iModulons that show similar activities across all the conditions in the dataset. Such clusters correspond to the classical definition of a Stimuli. Clusters with iModulons of interest were labeled on the right-hand side of the panel (marked in red). (**B**) iModulon activities within a cluster containing multiple CPS-associated iModulons in “CPS expression” conditions. The *x*-axis denotes RNA-seq conditions of strains engineered to express individual CPS (CPS1, CPS2, CPS4, CPS5, CPS6) and an acapsular strain lacking all CPSs [56]. The *y*-axis shows the activities of the corresponding CPS-associated iModulons (UpxYZ_CPS4, UpcYZ_CPS1, TF_CPS8, UpcYZ_CPS3, UpcYZ_CPS5, UpcY_CPS7, UpxYZ_CPS6) along with neighboring iModulons clustered in the vicinity. (**C**) Gene weight correlation between the UpxYZ_CPS6 and the BT0248 iModulon. Each dot represents a gene. The dashed lines indicate weight cutoff for iModulon membership. Orange dots represent significantly weighted genes shared between the two iModulons and denote shared regulatory targets. (**D**) An activity plot of the UpxYZ_CPS6 iModulon in selected conditions—“CPS expression” and “ECF-σ CRISPRi.”

To elucidate functional relationships between transcriptional modules under varying conditions, we performed hierarchical clustering analysis of iModulon activity profiles across 22 independent experiments encompassing 153 distinct conditions (Fig. 3A). Eleven previously uncharacterized ECF-σ-associated iModulons exhibited distinct activity patterns and clustered independently, reflecting the characteristic signaling diversity of ECF-σ factors [51].

The BtModulome effectively captured condition-specific activation patterns, with iModulon activities correlating with conditions that induce their constituent genes [17]. For example, cluster 4 comprised eight capsular polysaccharide biosynthesis (CPS)-associated iModulons displaying highly orthogonal activities in *B. thetaiotaomicron* variants expressing single CPS loci [9] (Fig. 3B). Unexpectedly, we observed moderate activation of the BT0248 iModulon in the *CPS6* variant, which includes BT0248 as the sole transcriptional regulator (ECF-σ). Comparative analysis revealed significant overlap between UpxYZ_CPS6 and BT0248 iModulons, particularly in genes encoding a transmembrane protein (BT0247), *cps6* gene subset, and putative lipoproteins (BT2987–BT2989), with BT0248 showing negative weight coefficients in the UpxYZ_CPS6 iModulon (Fig. 3C). The presence of genes in multiple iModulons indicates that their expression is regulated by multiple biological signals [17], which suggests the involvement of BT0248 in regulating CPS expression (Supplementary Note S7).

This observation yields two key insights. First, the negative gene weight coefficient of BT0248 suggested contra-regulation, a characteristic feature of transcriptional repressors [22, 52]. Supporting this hypothesis, CRISPRi-mediated repression of BT0248 enhanced UpxYZ_CPS6 iModulon activity (Fig. 3D), indicating that BT0248 negatively regulates both putative lipoproteins and *CPS6* genes, consistent with known ECF-σ-mediated regulation of multiple CPS loci (*CPS*1, 2, 3, 4, and 8) [53]. Second, while the putative lipoproteins (BT2987-BT2989) remain uncharacterized, their associations with CPSs in maintaining cell envelope integrity [54] and viral infection [55, 56 (Supplementary Fig. S9) suggest synergistic action with *CPS6* under ECF-σ regulation.

As such, this pairwise iModulon analysis enables functional interpretation of uncharacterized TFs through guilt-by-association principles [18]. The remaining novel ECF-σ-associated iModulons exhibited condition-specific responses to various stimuli and genetic contexts, including starvation, phage infection, co-culture, and global regulator inactivation (Supplementary Fig. S10). Below, we present detailed analyses of two previously uncharacterized ECF-σs.

### SigW-1 orchestrates transcriptional regulation of arylsulfatase expression

**Figure 4.**
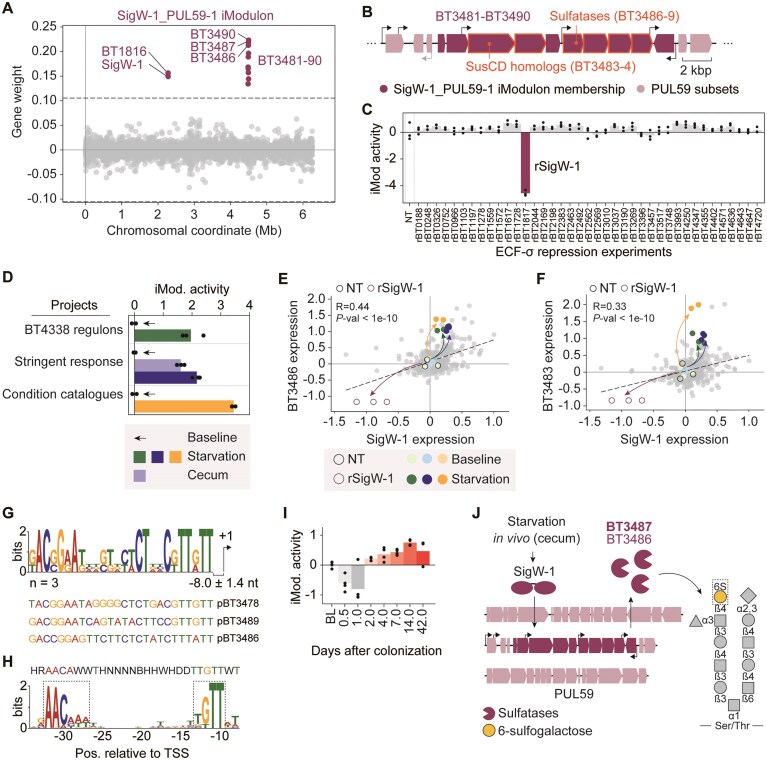
The SigW-1_PUL59-1 iModulon is regulated by SigW-1. (**A**) Scatter plot showing the gene weights within the SigW-1_PUL59-1 iModulon. Genes belonging to the iModulon are colored in purple, non-member genes are colored in gray. (**B**) The transcription architecture of BT3481–BT3490 operon. Promoters of interest are marked in black. (**C**) Activity of the SigW-1_PUL59-1 iModulon across ECF-σ repression experiments (*n* = 39). (**D**) iModulon activity under nutrient-depleted conditions and in cecum. The baseline control experiment in each project is indicated with an arrow. Comparison of gene expression between SigW-1 and (**E**) BT3486, and (**F**) BT3483 operon. NT: non-target control in ECF-σ CRISPRi experiments. (G, H) Motif analysis for (**G**) potential SigW-1 binding sites in panel (B). (**H**) Non-canonical promoters identified upstream of primary transcription start sites (TSSs) (Ryan *et al.*, 2020). Promoter motif was searched with MEME. The motif in panel (G) was non-significant (*P*-value > .05) (Supplementary Table S12). However, promoter sequence pBT3478 and pBT3489 showed distinct “TGTT” motif at approximately −10 of TSS, which matches that of the non-canonical promoter. (**I**) Changes in iModulon activity in days after colonization (from “*in vivo* adaptation” experiment). (**J**) Proposed mechanism and biological implications of SigW-1-mediated transcriptional regulation.

We identified a distinct iModulon, designated SigW-1_PUL59-1, that shows specific activation under “starvation-mimicking” conditions (Supplementary Fig. S10). This iModulon comprises a subset of PUL59, a locus activated by unidentified host dietary glycans [7], along with *sigW-1* (BT1817) and its associated transmembrane protein (BT1816) (Fig. 4A and B). Both gene clusters displayed positive gene weight coefficients, indicating coordinated activation in response to specific regulatory signals (Fig. 4A). CRISPRi-mediated repression of *sigW-1* significantly decreased iModulon activity, confirming SigW-1’s regulatory control over member genes (Fig. 4C). Conversely, the iModulon showed enhanced activity under starvation conditions and in the cecum compared to MM-glucose controls, identifying key activating signals (Fig. 4D).

Based on the regulatory role of SigW-1, we hypothesized its involvement in transcriptional control of the BT3481–BT3490 operon. Analysis of the published transcriptome dataset [57] revealed six TSSs, with internal TSSs upstream of BT3481 and BT3486 as likely regulatory targets (Fig. 4B). Gene expression analysis showed moderate positive correlations between SigW-1 and its putative regulons within the BtModulome (Pearson *R* = 0.44 and 0.33 for BT3486 and BT3483, respectively) (Fig. 4E and F; Supplementary Fig. S11). Importantly, significantly reduced regulon expression in the *sigW-1* repressed strain (hereafter rSigW-1) confirmed direct transcriptional control of SigW-1 over the PUL59 subset, while its enhanced expression under starvation conditions indicated nutrient-dependent activation (Fig. 4E and F).

Promoter sequence analysis revealed a potential SigW-1 binding consensus motif resembling a non-canonical promoter motif of *B. thetaiotaomicron* (Fig. 4G and H), typically associated with oxidative stress response genes and stationary phase-induced genes [57]. The regulons include BT3483 and BT3484, encoding SusC/D homologs-outer membrane transporters essential for polysaccharide utilization [58], and BT3486–BT3489, encoding four distinct sulfatases. These sulfatases facilitate nutrient acquisition by removing protective sulfated terminal caps from mucosal glycans [59], enabling nutrient foraging from mucosal *O*-glycans [7]. For this reason, sulfatases are important determinants of *in vivo* fitness and colonization, with broader implications in host-microbe communications and immunomodulation [60, 61]. Notably, arylsulfatase BT3487 specifically targets six-sulfogalactase in mucin glycans [59], suggesting direct involvement of the SigW-1_PUL59-1 iModulon in host-microbe interactions.

Consistent with the proposed role of sulfatases in colonization and host-microbe communication, the SigW-1_PUL59-1 iModulon showed progressive activation during *in vivo* adaptation of *B. thetaiotaomicron* (Fig. 4I) [62]. Consistent with these observations, transposon-inactivated mutants of *sigW-1* and the proposed regulons BT3481–BT3490 exhibited concurrent fitness reductions in post-colonization experiments in mouse intestines and in the presence of carbon sources found in mucosal glycans, including hyaluronic acid [45] (Supplementary Fig. S12). These findings establish SigW-1 as a key regulator of host adaptation, leading to our proposed model of its transcriptional regulation (Fig. 4J).

### SigH-1 mediates (p)ppGpp-dependent stringent response

**Figure 5.**
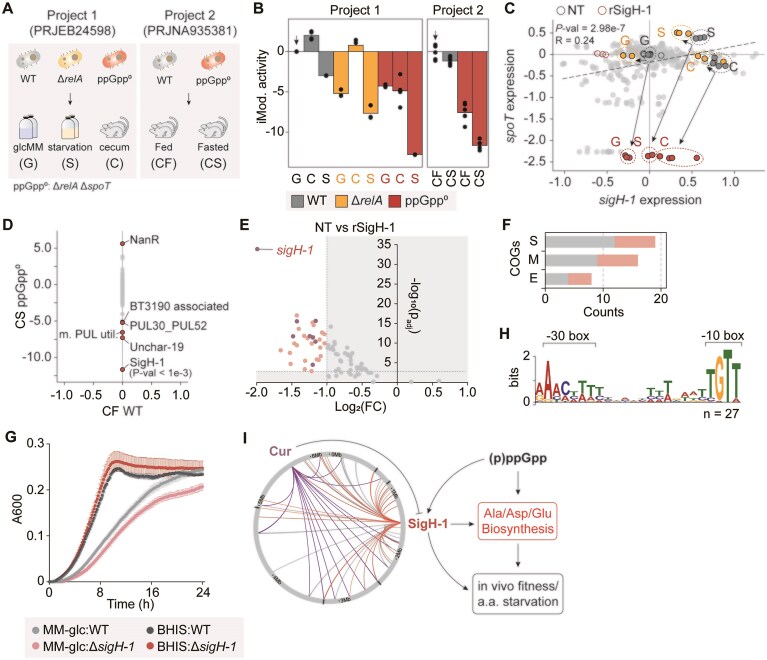
The SigH-1 iModulon is implicated with (p)ppGpp-mediate regulation in *B. thetaiotaomicron*. (**A**) Simplified experimental layout of two projects (PRJEB24598 and PRJNA935381) that showed variable SigH-1 iModulon activities. The first project contains RNA-seq of *B. thetaiotaomicron* WT, Δ*relA*, and ppGpp^0^ (Δ*relA* Δ*spoT*) in three different conditions: MM-glucose “G,” carbon starvation “S,” and mouse cecum “C” [10]. The second experiment involves WT and ppGpp^0^ strains inoculated in mice that are either fed “CF” and fasted “CS” [42]. (**B**) Activity profile of the SigH-1 iModulon in wild-type, Δ*relA*, and ppGpp^0^ background. The abbreviated labels on the *x*-axis are identical to those in panel (A). Baseline experiments are indicated with an arrow. (**C**) Gene expression profiles of *sigH-1* and *spoT* (BT3998) in the BtModulme. Color code is the same as in panel (A). (**D**) Differential iModulon activity graph between wild-type fed *in vivo* (CF WT), and ppGpp^0^ mutant fasted *in vivo* (CS, ppGpp^0^). m. PUL util.: multi-polysaccharide utilization. (**E**) DEG [1 > Log_2_(fold-change) < -1, P_adj_ < 0.01) analysis in rSigH-1 relative to NT control. Dots shown in light pink represent DEGs, dark pink represent differentially expressed *cur* regulons, while rest were marked in gray. (**F**) Selected COG categories of genes in the SigH-1 iModulon and the proposed *sigH-1* regulons. Color code is identical to panel (E). COG abbreviations—S: unknown, M: cell wall/membrane/envelope biogenesis, E: amino acid transport and metabolism. (**G**) Growth profile of the *B. thetaiotaomicron* wild-type and ΔSigH-1 in BHIS and MM-glc liquid media. (**H**) Conserved promoter motif identified in the SigH-1 regulons. Promoter motif was searched by MEME (zoops, *P*-value < .05). (**I**) Proposed regulatory network and functional association between Cur, SigH-1, and (p)ppGpp.

We discovered an ECF-σ-associated SigH-1 (encoded by BT1197) iModulon exhibiting drastic activity changes in response to mutations in *cur, spoT*, and *relA* (Fig. 5A and B; Supplementary Fig. S13A). These genes encode Cur, a master regulator that orchestrates the expression of more than 170 genes involved in *in vivo* colonization, carbohydrate utilization, and antibiotic susceptibility [12, 50, 63], and RelA/SpoT homologues, which synthesize the stringent response alarmone (p)ppGpp. Thus, such changes in the iModulon activity suggests potential links between iModulon members and stress-adaptive responses in *B. thetaiotaomicron* [10].

Interestingly, 21 of the 79 members of the SigH-1 iModulon were identified as Cur regulons (Supplementary Fig. S13B and Supplementary Table S13) [8]. This seems to account for the significant increase in iModulon activity in Δ*cur* background and suggests that these gene members are likely subject to negative regulation by Cur. Similarly, SigH-1 expression negatively correlates with Cur (Pearson *R* = −0.32; Supplementary Fig. S13C), with Cur deletion significantly upregulating *sigH-1* expression but not vice versa. This hierarchical regulation, confirmed by previous ChIP-seq data [8], establishes Cur as a SigH-1 repressor.

In contrast, the SigH-1 iModulon exhibited markedly reduced activity in (p)ppGpp-deficient (ppGpp^0^) strains (Fig. 5B and C; Supplementary Fig. S13D). Given that (p)ppGpp modulates transcriptional programs through σ factor competition and ECF-σ activations [64, 65], the results suggest (p)ppGpp-dependent regulation of *sigH-1* (Fig. 5C). This regulatory connection is particularly significant as (p)ppGpp deficient *B. thetaiotaomicron* shows severe growth defects in its native niche, failing to propagate during feast-famine cycles and being outcompeted by wild-type counterparts in the mouse intestine [10, 42]. ICA revealed that the SigH-1 iModulon showed the most pronounced activity changes between wild-type and ppGpp^0^ strains during intestinal feast-famine cycles (Fig. 5D and Supplementary Fig. S13E and F). This indicates that SigH-1 iModulon members may substantially influence transcriptional responses to (p)ppGpp deficiency during intestinal adaptation. Taken together, considering Cur’s role as a global regulator that mediates multiple gene responses and the function of (p)ppGpp in stringent responses, we hypothesized that SigH-1 mediates functionally similar gene effector responses in *B. thetaiotaomicron*.

To identify SigH-1 regulons, we performed differential gene expression analysis between the *sigH-1* repressed strain (hereafter rSigH-1) and the control strain, identifying 31 significantly regulated genes (|Log_2_FC|${\mathrm{\ }} \ge $ 2; p_adj_  $ \le $ 0.01; Fig. 5E). COG analysis revealed enrichment in unknown functions (COG S), cell wall/membrane/envelop biogenesis (COG M), and amino acid transport/metabolism (COG E) (Fig. 5F and Supplementary Fig. 13G). The COG E category included key enzymes in L-alanine and L-glutamate metabolism: aspartate decarboxylase (BT0735), glutamate decarboxylase (BT2570), glutaminase (BT2571), and a permease (BT2567). The SigH-1 iModulon also includes additional amino acid biosynthetic genes such as aspartate ammonia lyase (BT2755) and an asparaginase II (BT2757), which showed moderate downregulation in rSigH-1 strain (Supplementary Table S13). Such regulon profiles overlap with the alanine/aspartate/glutamate metabolic pathway downregulated in ppGpp^0^, which exhibited slower growth under amino acid limitation compared to wild type [10]. Similarly, the Δ*sigH-1* mutant exhibited reduced cell density in amino acid-restricted MM-glc media, but not in an amino-acid rich complex medium (Fig. 5G and Supplementary Fig. S13H and I). To further characterize the rSigH-1 mediated effects, we performed genome-wide DEG analysis. Repression of *sigH-1* did not result in expression changes in Cur regulons associated with carbohydrate utilization and gut colonization (Supplementary Fig. S14A and B). In addition, whereas the ppGpp^0^ exhibited significant down-regulation of genes within the α-ketoglutarate biosynthetic module [10], rSigH-1-mediated changes were not statistically significant. However, pyruvate carboxylase subunit B (BT1196), which converts pyruvate to oxaloacetate, did show a significant decrease in the rSigH-1 strain (Supplementary Fig. S15A). Despite these differences, the fold-change profiles of genes significantly altered in the ppGpp⁰ background [10] showed moderate correlation with those in the rSigH-1 strain. This correlation strengthened under stress conditions: Pearson’s *R* increased from 0.30 in control conditions (G) to 0.43 under starvation (S) and 0.46 during *in vivo* colonization (C) ( Supplementary Fig. S15, B, C, D and Supplementary Table S14). These findings suggest that SigH-1 functions as an ECF-σ “stress relay,” integrating Cur-mediated repression with (p)ppGpp-dependent activation. This integration appears to orchestrate amino acid biosynthesis (alanine/aspartate/glutamate) and other stress response genes (Supplementary Tables S13 and S14) that likely contribute to survival and competitive fitness during nutrient limitation and *in vivo* colonization.

Promoter analysis identified a distinct −30/−10 box resembling the non-canonical motif of *B. thetaiotaomicron* in SigH-1 regulons (Fig. 5H). Previous report linked such alternative promoters to oxidative stress response genes activated during stationary growth phase [57], consistent with the proposed role of *sigH-1* in starvation responses and (p)ppGpp-mediated regulation. Based on these findings, we present an integrated model of SigH-1 regulatory network and its stress-response functions (Fig. 5I).

## Discussion

Here, we present BtModulome, a comprehensive transcriptional regulatory framework derived from ICA decomposition of large-scale *B. thetaiotaomicron* transcriptome data. The framework comprises 110 independently modulated gene sets, called iModulons, explaining 72.9% of transcriptome variance across 153 distinct RNA-seq conditions. Approximately 74% of these modules show functional coherence and evidence of common regulatory control, enabling knowledge-based interpretation of ICs derived from unsupervised machine-learning algorithms. The benefit of iModulons lies in its ability to infer novel transcriptional regulatory associations and provide deeper understanding of cellular functions. For example, BtModulome has shed light on the presence of multiple, independent regulations acting within PULs, identified a group of genes with a potential role in bile salt-specific responses, and novel associations of ECF-σ in mediating diverse cellular responses.

Our analysis particularly illuminated the regulatory roles of ECF-σ factors. Through systematic analysis of iModulons exhibiting significant activity changes in response to CRISPRi-mediated perturbation of ECF-σ factors, or those including ECF-σ factors in their membership, we uncovered regulatory networks for 11 previously uncharacterized ECF-σ factors. Integration of iModulon activity with multi-omics datasets revealed diverse biological roles, including stress response (SigR-1 and SigH-1), nutrient acquisition (SigW-1), and capsular polysaccharide regulation (BT0248). Interestingly, motif analysis showed that the SigW-1 and SigH-1 regulon promoters closely match the −30/−10 non-canonical motif previously described in *B. thetaiotaomicron* [57]. The same study demonstrated that genes driven by this non-canonical element are enriched for oxidative-stress responses and are induced during stationary phase [57]. Here, we present evidence that these non-canonical promoters may act as recognition elements for ECF-σ factors, including SigW-1 and SigH-1. While seven ECF-σ iModulons remain functionally ambiguous, reflecting challenges in studying non-model organisms, our findings significantly expand understanding of stress response mechanisms in *B. thetaiotaomicron*.

The ECF-σs characterized in this study align with phylogenetic classifications from ECFhub [28], which groups ECF-σs based on protein sequences and regulation mechanisms. For instance, SigR-1 and SigH-1 belong to the ECF114 family associated with aerotolerance and oxidative stress protection [66]. The regulatory hierarchy of SigR-1 in the PerR iModulon and its role in stress responses align with this classification. While other SigR analogs regulate thiol-oxidative stress [49], our findings suggest that SigR-1 in *B. thetaiotaomicron* has evolved distinct functional roles. Similarly, SigH plays multifaceted roles in mediating bacterial stress responses from heat shock, thiol-oxidative stress responses, and redox metabolism [66–68]. Our analysis revealed that putative thioredoxins and multiple efflux pumps co-expressed with SigH-1 (Supplementary Table S13), suggesting a potential role for SigH-1 in mediating redox stresses in *B. thetaiotaomicron*. We also show that SigH-1 (BT1197) regulates amino acid metabolism in association with the (p)ppGpp stress response cascade, which is unforeseen in other SigH analogs and other ECF114 sigma factor families. The course grained analysis of iModulon activity offered insights into the complex interplay between SigH-1 and other transcription regulators. Notably, the major transcriptome changes in ppGpp^0^, which exhibited significant fitness defects in mouse intestines [10], were attributed to the SigH-1 iModulon regulated by Cur and SigH-1. This illustrates intricate transcription networks involving multiple regulators in mediating responses to stress and *in vivo* fitness.

Beyond stress responses, we demonstrate transcriptional regulation of PUL genes by the distant ECF-σ, SigW-1 (BT1817). iModulon analysis showed that SigW-1 regulates a subset of PUL59 that encodes multi-copies of sulfatases. This finding challenges the conventional understanding that PULs typically contain their own ECF-σ/anti-σ pairs [7]. SigW-1 belongs to the poorly characterized ECF251 family. The only known member is SigE, which regulates oxidative stress and virulence network in a pathogenic bacterium *Porphyromonas gingivalis* [69]. Our analysis reveals the unexpected role of SigW-1_PUL59 in *in vivo* strain fitness, rather than oxidative stress and virulence. While a previous report suggests the involvement of a *B. thetaiotaomicron* sulfatase in eliciting inflammatory response in hosts [60], it remains unclear whether the sulfatases encoded by BT3486–3489 play similar roles.

Lastly, sensitivity analysis of CRISPRi-based ECF-σ perturbations highlighted their importance in resolving transcriptional modules that are otherwise invisible in a compendium of native conditions, as exemplified by BT0248 and *sigW-1* (Supplementary Fig. S16 and Supplementary Note S8), highlighting the important utility of targeted gene perturbation datasets in refining novel regulator–regulon relationships. The complete BtModulome, including iModulon genes, activity metrics, and experimental conditions (Supplementary Tables S6–S8), provides a valuable resource for exploring additional regulatory modules and represents a foundation for hypothesis-driven research in gut microbe biology. Beyond biological discovery, the BtModulome has practical applications in strain engineering. Recent studies have demonstrated that utilizing entire genes in iModulons, including hypothetical genes, as functional modules translate to more effective phenotypic outcomes compared with traditional gene-centric approaches [20, 21]. The modular nature of iModulons particularly benefits engineering efforts in non-model organisms by enabling systematic exploitation of uncharacterized genes.

Future studies should be focused on addressing uncharacterized iModulons, which reflects both limited functional annotation in non-model organisms and technical constraints of ICA decomposition. While extensive research has investigated *B. thetaiotaomicron* PULs [7, 43, 53, 70–73], expanding the diversity of high-quality transcriptome data will further refine understanding of its regulatory networks. Furthermore, inclusion of small noncoding RNAs, which have recently been shown to mediate various aspects of *B. thetaiotaomicron* physiology, including carbon metabolism [57, 74], and sensitivity toward antibiotics [50] and bile acids [75], in the iModulon framework merits further investigation.

## Supplementary Material

gkaf1166_Supplemental_Files

## Data Availability

The iModulon metadata (JSON file) is available in Zenodo (DOI: 10.5281/zenodo.17015224). Transcriptome sequencing data generated in the current study have been deposited in the EMBL Nucleotide Sequence Database (ENA) with primary accession number PRJEB86907.
