# Supplementary material for: Transcriptional regulatory networks of the human gut symbiont Bacteroides thetaiotaomicron are uncovered using machine learning
Source: Nucleic Acids Res. 2025 Nov 6;53(20):gkaf1166. doi: 10.1093/nar/gkaf1166 (PMC12596196; doi:10.1093/nar/gkaf1166)
Supplement: gkaf1166_Supplemental_Files [file gkaf1166_supplemental_files.zip › SI_BtiModulon_NAR_R2.pdf]

SUPPLEMENTARY INFORMATION FOR

Transcriptional regulatory networks of the human gut symbiont *Bacteroides thetaiotaomicron* are uncovered using machine-learning

AUTHORS

Kangsan Kim,[1,2] Donghui Choe,[3] Sun Chang Kim,[2] Sung Sun Yim,[1,2,5] Ki Jun Jeong,[4,5] Bernhard Palsson,[3] Suhyung Cho,[2]* Byung-Kwan Cho[1,2,5]*

[1]Department of Biological Sciences, Korea Advanced Institute of Science and Technology, Daejeon 34141, Republic of Korea

[2]KI for the BioCentury, Korea Advanced Institute of Science and Technology, Daejeon 34141, Republic of Korea

[3]Department of Bioengineering, University of California San Diego, La Jolla, CA 92093, USA

[4]Department of Chemical and Biomolecular Engineering, Korea Advanced Institute of Science and Technology, Daejeon, 34141, Republic of Korea

[5]Graduate School of Engineering Biology, Korea Advanced Institute of Science and Technology, Daejeon, 34141, Republic of Korea

* To whom correspondence should be addressed. Email: bcho@kaist.ac.kr (B.-K.C.) and shcho95@gmail.com (S.C.)

This PDF file includes:

Supplementary Notes S1 to S8

Supplementary Table S12

Supplementary Figures S1 to S16

Reference

SUPPLEMENTARY NOTE

Supplementary Note S1. Implementing dCas9-based gene repression system in *B. thetaiotaomicron.*

To generate the compendium of RNA-Seq data on individual ECF-σ-repressed strains, we used a CRISPR interference (CRISPRi) approach that leverages the highly efficient gene knockdown (1-4) in *B. thetaiotaomicron*.

We first validated whether our dCas9-based CRISPRi setup could recapitulate the efficient gene knockdown described previously (1). To ensure stable expression of the repression machinery, we genomically integrated dCas9 in-frame with BT0418, a locus encoding an outer membrane porin with consistently high expression across different laboratory conditions (**Supplementary Fig. S1A** and **Supplementary Table S3**). We confirmed inducible dCas9 expression at a working inducer (IPTG) concentration of 100 μM (**Supplementary Fig. S1B**).

Next, we validated functional expression of the dCas9–sgRNA complex by genomically integrating an expression construct encoding an sgRNA targeting superfolder GFP (sfGFP) (5), which is co-expressed within the same construct, into the serine tRNA loci BTt71 and BTt70, respectively (**Supplementary Fig. S1C**).

To optimize sgRNA expression, we tested four promoters with varying expression strengths (6). In this library, sfGFP expression was driven by either a strong promoter (PBfP1E6, "Sf1") or a weaker promoter (PcfiA, "Sf2") to account for reporter gene promoter context, while sgRNA was expressed from one of four promoters of descending strength (PBfP1E6, PBfP4E5, PBfP5E4, PBT1311, denoted S1, S2, M1 and M2, respectively). This design yielded eight ORF-targeting ("Target") and eight non-targeting ("NT") control strains, each uniquely defined by its sgRNA:sfGFP promoter pairing. (**Supplementary Fig. S1C**).

We then measured sfGFP fluorescence in each strain in BHIS liquid medium supplemented with or without 100 μM of IPTG. (**Supplementary Fig. S1D**). In the strong sfGFP (Sf1) series, basal (IPTG-) repression was already detectable (fold reduction vs NT: 0.66× for S1, 0.86× for S2, 0.88× for M1 and 0.73× for M2), attributable to 'leaky' dCas9 expression (**Supplementary**

Fig. S1B). IPTG induction further enhanced knockdown to 0.48× (Sf1_S1), 0.74× (Sf1_S2), 0.79× (Sf1_M1) and 0.69× (Sf1_M2) of the NT control. In contrast, the weak-sfGFP (Sf2) series exhibited only marginal repression upon induction (**Supplementary Fig. S1D**). These results showed effective target gene repression tunable by promoter strength.

To further characterize the dynamics and robustness of our CRISPRi system, we measured dose- and time-dependence of sfGFP knockdown using the Sf1_S1_ORF strain, which exhibited the most effective target gene knockdown. First, we titrated IPTG from 0 to 500 μM – the maximum concentration used in the previous report (1), and quantified fold reduction in sfGFP fluorescence relative to the NT control (**Supplementary Fig. S1E**). We observed gradual fluorescence reduction that reached 0.58× at 100 μM and plateaued between 250-500 μM (0.51×), confirming that 100 μM IPTG is sufficient for target gene repression.

We then assessed temporal stability of repression by sampling fluorescence at 6, 8, 12, and 24 h post-induction, covering early-, mid-exponential and stationary growth phase (**Supplementary Fig. S1F**). The fold reduction in RFU/A600 was approximately 0.62× as early as 6h, reached approximately 0.46× by 12h, and remained at that level through stationary phase, confirming sustained gene knockdown throughout the growth phase (**Supplementary Fig. S1G**).

Finally, we tested whether the gene repression translates in different contexts: (i) native genes with varying expression strengths (ECF-σs) and (ii) Columbia blood agar culture (CBA). We selected three ECF-σs – BT1197 (SigH-1), BT1728 (SigL-3), and BT1817 (SigW-1) – representing high, medium, and low gene expression among the active ECF-σs (**Supplementary Fig. S2A**), respectively (**Supplementary Fig. S1H**). Each ECF-σ was targeted by an sgRNA driven from the strong P_BfP1E6 promoter. Strains were plated on CBA agar containing 100 μM IPTG to induce dCas9 expression and harvested for RNA extraction. qRT–PCR quantification of ECF-σ transcript levels revealed target-dependent knockdown: SigH-1 (highly expressed) was reduced to 0.39× wild-type levels, and SigL-3 and SigW-1 transcripts were more strongly repressed (0.11× and 0.07×, respectively) (**Supplementary Fig. S1I**). Together, these results demonstrated that the CRISPRi system functions robustly across diverse genomic contexts and culture settings.

Supplementary Note S2. Design, construction and validation of a CRISPRi library targeting active ECF-σs in *B. thetaiotaomicron*.

Among the 50 ECF-σs annotated in the *B. thetaiotaomicron* genome (7), we first evaluated the baseline expression of all 50 annotated ECF-σ factors in *B. thetaiotaomicron* grown on CBA agar by analyzing RNA-Seq data (**Supplementary Fig. S2A**). Of these, we designated the top 75% of the distribution (above the first quartile, Q1) as 'active' and selected the 41 ECF-σ genes in this category for functional repression.

To target each of these 41 ECF σ-factor genes, we used the CRISPOR platform to design 20-nt protospacer sequences (8), which were cloned by T4 DNA ligation into the pMM553 backbone under the synthetic PBfP1E6 promoter (**Supplementary Fig. S2B**). Each sgRNA plasmid was conjugated into a *B. thetaiotaomicron* strain constitutively expressing LacI-dCas9 from the BT0418 locus, yielding 41 independent CRISPRi strains. We then collected the total RNA from each strain cultured on CBA with 100 µM IPTG and performed RNA-Seq (**Supplementary Fig. S2B**). Differential expression analysis (DESeq2) against a non-targeting control revealed that 39 of 41 target genes were effectively repressed ($\log_2$(FC) < 0). Two guides targeting BT1053 and BT4705 failed to decrease transcript levels ($\log_2$(FC) > 0) and were excluded from downstream analysis (**Supplementary Fig. S2C**). Note that the housekeeping gene BT1311 remained unchanged across all 41 strains (**Supplementary Fig. S2D**), confirming that knockdown was specific to the intended ECF-σ targets and did not arise from off-target effects or global transcriptional perturbation.

Supplementary Note S3. Construction of *B. thetaiotaomicron* RNA-Seq compendium.

As iModulon analysis fundamentally represents a signal-separation problem, we therefore increased transcriptome diversity by combining in-house and publicly available RNA-Seq datasets with CRISPRi-repressed ECF-σ RNA-Seq profiles. To this end, we searched and downloaded relevant datasets using the SRA-toolkit (see **Methods**). This yielded a total of 336 independent RNA-Seq samples derived from 20 independent projects (**Supplementary Table S5)**. Importantly, the public RNA-Seq database covers an array of different niche-specific (e.g. *in vivo*, co-culture) (9-15), carbon-specific (16-21), and stress-specific

(9,11,15,16,22-24) conditions and genetic backgrounds (e.g. gene deletion) (9,13,22-24) that may potentially invoke ECF-σ-mediated gene regulation. Lastly, we added eight additional in-house generated RNA-Seq datasets that reflect various culture conditions, including minimal medium supplemented with glucose (MM-glc), mucin (MM-muc), and two complex media including BHIS and CBA. Together, this yielded *B. thetaiotaomicron* RNA-Seq compendium containing 461 independent RNA-Seq datasets.

Supplementary Note S4. PUL14 can be explained by three distinct iModulons.

ICA predicted that three independently-regulated operons exist in PUL14 (**Supplementary Fig. S5, A** and **B**), which encodes saccharolytic enzymes that are induced in the presence of porcine mucosal glycans (25). Each of the three iModulon sub-groups of PUL14 contains a set of SusC and SusD homologs that are typically involved in binding and transport of complex carbohydrates (26), further suggesting the presence of distinct, substrate-dependent regulations acting on the PUL14 (**Supplementary Fig. S5B**). In fact, the three iModulons demonstrated different activities in response to mucin, *N*-glycans, and in a carbon-depleted environment (**Supplementary Fig. S5C**), which seems to be in keeping with the previous observations that SusCD systems are induced by different TFs in a substrate-specific manner (25).

Supplementary Note S5. Assessment of fitness and biofilm formation capacity in gene knockout mutants in response to bile salts.

To test the functional relevance of the gene members in the Bile salt efflux iModulon, we assessed the bile-induced stress responses in each of the seven knockout mutants, including ΔBT2792-2795, ΔBT1964-1967, ΔBT2940-2942, ΔBT0691-0692, ΔBT1789-1799, ΔBT2117-2119, and ΔBT3822 (**Supplementary Fig. S6**). Earlier studies characterized bile-dependent phenotypic changes in *B. thetaiotaomicron* by means of strain fitness (27) (specific growth rate) and the biofilm formation capacity (28).

First, the changes in fitness of each knockout mutant in the presence of bile salts demonstrated, consistent with the previous Tn-Seq study, a pronounced reduction in growth rates in ΔBT2792-2795 and in ΔBT0691-0692, while the others remained similar to the wild-type (**Supplementary Fig. S6, A** and **B**) (27). Next, we assessed bile-induced biofilm formation capacity of *B. thetaiotaomicron* (28) in each knockout mutant. Previously, the genetic determinants of biofilm formation reported in *B. thetaiotaomicron* included capsular polysaccharide biosynthesis clusters (29) and a type V pilus (30), which led to an increase in biofilm formation capacity upon inactivation or stand-alone expression. More recently, deletion of *bipABC* which encode Resistance-Nodulation-Division (RND) efflux pump transporting magnesium divalent cations in the biofilm matrix, led to impaired bile-induced biofilm formation in *B. thetaiotaomicron* (31). Unexpectedly, a significant reduction in biofilm formation capacity was observed in ΔBT0691-0692, but not in other RND efflux pumps (**Supplementary Fig. S6C**). BT0691 and BT0692 are annotated as DUF3575 domain-containing outer membrane protein, and calcineurin-like phosphohydrolase domain-containing cytosolic protein, respectively. While the mechanistic role that BT0691-0692 plays in biofilm formation remains unclear, the module-driven inference of gene functions enabled identification of the potential genetic determinant of biofilm formation in *B. thetaiotaomicron*.

Supplementary Note S6. The 'closest match' iModulons contain multiple PULs and carbohydrate-active enzymes.

The regulatory iModulons with low precision and recall are the result of retaining only a subset known regulons of the enriched regulator(s), while containing larger proportion of genes that are not part of the known regulons (32). Examples are three PUL iModulons designated as 'multi polysaccharide utilization (MPU)', 'multi polysaccharide utilization-2 (MPU-2)', and NanR (**Supplementary Table S10**). Each iModulon contains more than one PUL, along with multiple carbohydrate active enzymes (CAzy), catalytic enzymes, and unknown proteins. The fact that a single type of complex carbohydrate could elicit activation of multiple PULs, and that degradation of polysaccharides requires a concerted action of multiple enzymes (19,25,33,34), it can be deduced that the three iModulons represent gene

sets that are co-activated in response to *in vivo* cultivation and the presence of mucosal glycans (**Supplementary Fig. S7**). Another consideration is that the MPU iModulon, for instance, contains subset of genes that belong to 7 different PULs, including those saccharolytic to pectic glycans, arabinogalactans, other host glycans, and unknown substrates (**Supplementary Table S11**). The BtModulome inherently lacks transcriptome data that reflect such dietary substrate-specific transcriptome responses. Since the signal deconvolution performance of ICA is known to improve in proportion to the variance of signal strengths between samples (35), the relative paucity of substrate-specific RNA-Seq data may have led multiple PULs to be identified as an independent component. Thus, it should be noted that the 'closest match' iModulons may also be the result of technical noise rather than a biological phenomenon (36).

Supplementary Note S7. BT0248 shows signs of contraregulation on CPS6 expression.

To investigate potential regulatory interplay, we analyzed the correlation of gene weight vectors between two iModulons – UpxYZ_CPS6 and BT0248, an approach used previously to reveal relationships between independent transcriptional modules (37). In an iModulon framework, genes with large positive or negative weights in a given component are considered members of that iModulon's regulon. Therefore, if two iModulons share a set of significantly weighted genes, it implies that the underlying regulators or signals have overlapping targets.

In our analysis, the BT0248-associated iModulon exhibits a notable overlap with the UpxYZ_CPS6 iModulon (**Fig. 3C**), meaning several genes are strongly weighted in both. Genes falling in the same quadrant have concordant weight signs (co-regulated in the same direction in both iModulons), whereas genes in opposite quadrants have opposing weight signs (suggesting inverse correlation between gene expression and iModulon activity). For example, BT0248 shows a high positive gene weight on the x-axis (BT0248 iModulon) but a negative weight on the y-axis (UpxYZ_CPS6 iModulon), indicating that BT0248 expression is inversely associated with CPS6 iModulon activation. The overlap of significantly weighted genes between these iModulons, including BT0248 and several CPS6 locus genes, suggests that BT0248 is involved in CPS6 regulation.

Supplementary Note S8. Sensitivity of iModulon reconstruction to removal of ECF-σ repression datasets.

We performed a sensitivity analysis by re-running ICA after removing all CRISPRi RNA-Seq datasets corresponding to four representative ECF-σ factors: SigR-1 (BT1572), SigH-1 (BT1197), BT0248, and SigW-1 (BT1817). Gene weights from the reduced models were compared to those in the full BtModulome (**Fig. S16**). This analysis revealed two distinct patterns.

(1) Robust modules buffered by regulatory hierarchy: Removal of SigR-1 and SigH-1 datasets had negligible effects (Pearson $r > 0.97$). Their iModulons were preserved because these σ factors are embedded in broader hierarchical networks – SigR-1 within the PerR oxidative stress regulon, and SigH-1 downstream of Cur and (p)ppGpp stringent response. Thus, their signals were redundantly encoded in other conditions (**Fig. S16, A** and **B**). This demonstrates that the iModulon is generally robust to replicate removal and not disproportionately driven by single experiments, provided that the transcriptional regulatory network (TRN) of interest is represented within the existing RNA-Seq compendium.

(2) Perturbation-dependent modules: In contrast, removal of BT0248 repression data abolished the contra-regulatory structure linking BT0248 with the cps6 cluster (BT1705–25) and lipoproteins (BT2987–2989), while removal of SigW-1 datasets effectively masked majority of the SigW-1_PUL59 iModulon members (Pearson $r = 0.11$) (**Fig. S16, C** and **D**). These relationships were uniquely captured when the corresponding CRISPRi perturbations were included, highlighting that direct gene perturbation method can complement for the absence of cognate environmental cues required to induce or repress target gene expression.

Importantly, even for ECF-σs embedded in hierarchical or overlapping networks (e.g., SigR-1 and SigH-1), the corresponding repression datasets are not dispensable. Rather, they provided critical differential expression evidence (via DESeq) that validated and refined potential regulons within their iModulons (**Fig. S8C** and **Fig. 5E**). Together, this analysis highlights the essential role of CRISPRi RNA-Seq datasets in resolving condition-specific or repressive regulatory interactions within the BtModulome.

SUPPLEMENTARY TABLE

Supplementary Table S12. The top enriched motif of TF/σ-factor regulons identified in literature and in iModulon. Regulators whose cognate regulons contained at least six independent sequences and whose motifs scored *p-val* < 0.05 are shown.

Regulator locus	Name	Enriched motif	E-val./ mode	Sites	Width
BT0215	PerR	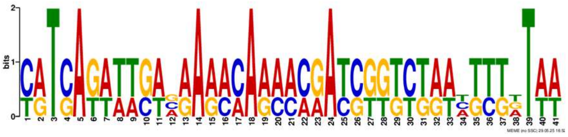<br>YRTSAKWWWSWVRARMMARMMMRATSKKTYTRWHTTTDTWW	$7.8e^{-16}$ /zoops	6/16	41
		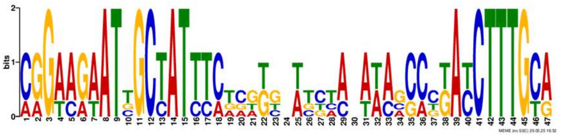<br>CGGAAGAATTGCTATTTCWVDKBNWTBWANAWAVCCBKAYCTTTGCA	$8.3e^{-8}$ /zoops	4/16	47
BT0267	HTCS_Aga-1	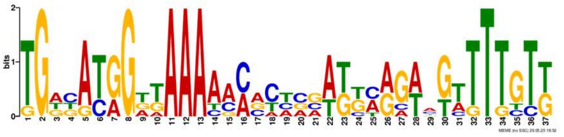<br>TGDBATGGDKAAAAMMVYHVVAKKMRGWMGWTTTGTK	$2.1e^{-14}$ /zoops	7/12	37
BT0366	HTCS_Ara-1	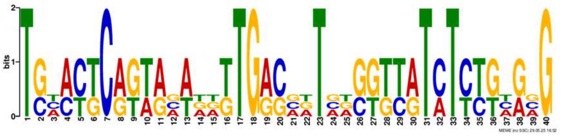<br>TSHMYKCRKWRVWDDKTGRSVDTVDSKKTRTMTYTKHRVG	$4.8e^{-17}$ /zoops	6/8	40
BT0433	NanR	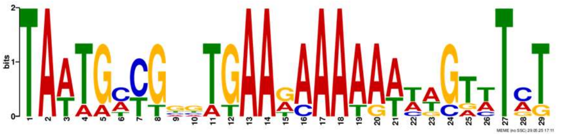<br>TAWTGMYGGGTGAARAAAAAWWWGTWTMT	$1.6e^{-9}$ /oops	6/6	29
BT0596	UpcY_CPS3	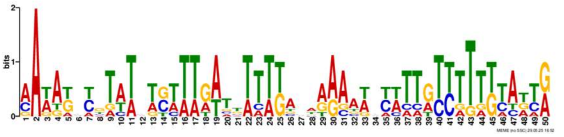<br>AAWAKHYKTWTHWSWTTRATKTTTKANWAAAAWDYWTTRTYTTTKTADTR	$1.0e^{-3}$ /zoops	7/7	50

BT1052/<br>BT1053	ECF_PU<br>L14	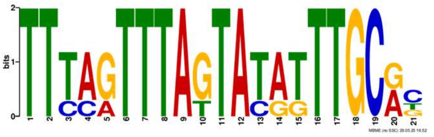<br>TTTAGTTTAGTATATTTGCRB	$2.2e^{-3}$<br>/zoops	4/9	21
BT1197	SigH-1	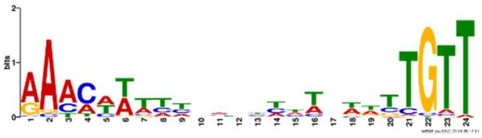<br>AAACWWWWYYNHNHHHWNWWTTGTT	$2.8e^{-56}$<br>/oops	61/61	24
BT1357/<br>BT1358	UpxYZ_<br>CPS4	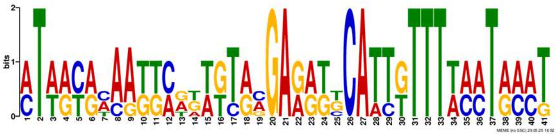<br>MTWRYRHAAKKMDDWKTRVGARRKKCATTKTTTWMMTRM<br>MT	$2.5e^{-10}$<br>/zoops	5/13	41
BT1754	HTCS_F<br>ru	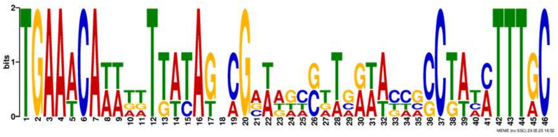<br>TGAAACAWWDDTTATAGNCGVWDDHSDTVRWABHVCCTA<br>HMTTTGC	$2.8e^{-7}$<br>/zoops	4/8	46
BT1817*	SigW-1	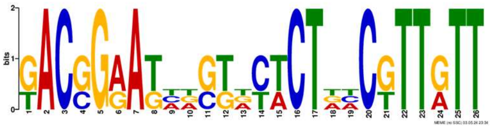<br>KACSGRAKHDSKDYWCTDHCKTTRTT	$3.4e^{-0}$<br>/zoops	3/6	26
BT2169	ECF_PU<br>L27	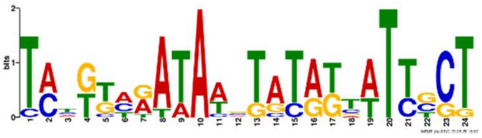<br>TMWKKHRATAWVTATAKTWTTBCT	$2.7e^{-9}$<br>/zoops	9/9	24
BT2511	Zur	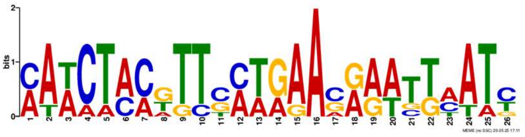<br>MAWCTAMKTTSMWRAASRAWTKMATY	$1.2e^{-3}$<br>/oops	6/6	26
BT2569	SigH-2	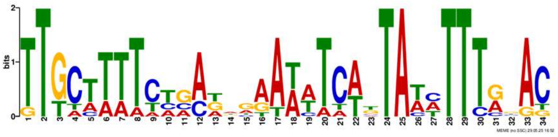<br>TTGCTTTTCWVADDRAAWWTCWKTAWHTTTRBAC	$1.1e^{-9}$<br>/zoops	8/15	34
BT2628	HTCS_<br>Man-1	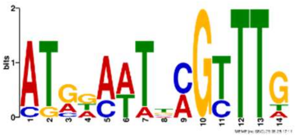	$8.3e^{-3}$<br>/oops	7/7	14

		ATRDAWTKMGTTTG			
BT2792	BT2792	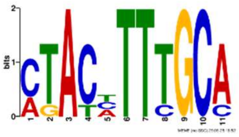 MTACHTTTGCA	2.9e^{-4} /zoops	6/7	11
BT3517	ECF_PUL60	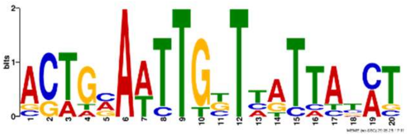 ACTGVAWTTGBTHATTAYMT	6.1e^{-8} /oops	8/8	20
BT3609	AraC_PUL62	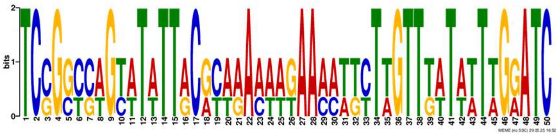 TCSGSYSWGYWTWTTRCRYWRAMWWKAAMMWKYTWGTTKWTWWTWGRATC	1.3e^{-7} /zoops	3/7	50
BT3786	HTCS_Man-3	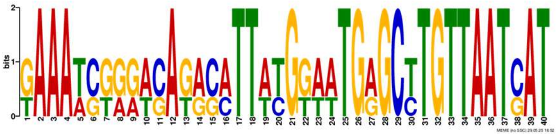 KAAAWSKRRWSAKASATTWTGKWWTGAGCTTGTTAATSAT	6.5e^{-5} /zoops	3/15	40
BT4178/BT4182	HTCS_Rgu-2b/a	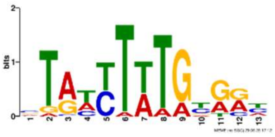 VTRWYTWTGYRRH	4.3e^{-2} /zoops	19/19	13
BT4338	Cur	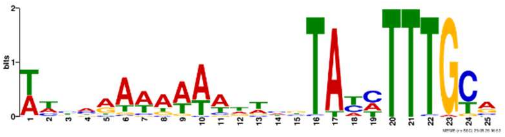 WWBMRAWAAAAWWHBTAYMTTTGCR	4.4e^{-62} /zoops	44/111	25
		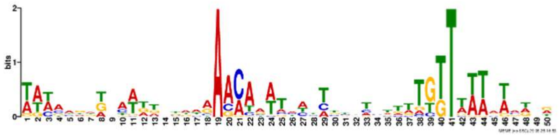 WWHAHDDDNMWDKNKDWRAAMAAWTHHHTBYHTNWTWTGTTWWWRWVDND	3.8e^{-21} /zoops	28/111	50
BT4720	Das1	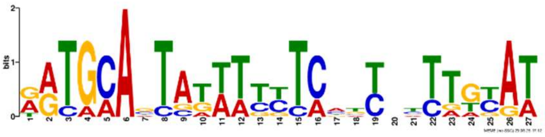 RRTGCASTATWTTYTCMKYHYTTKYAT	3.7e^{-6} /zoops	7/7	37

*Enriched motif in SigW-1 is analyzed using the promoters identified in Fig. 4B and visualized despite non-significance.

SUPPLEMENTARY FIGURE

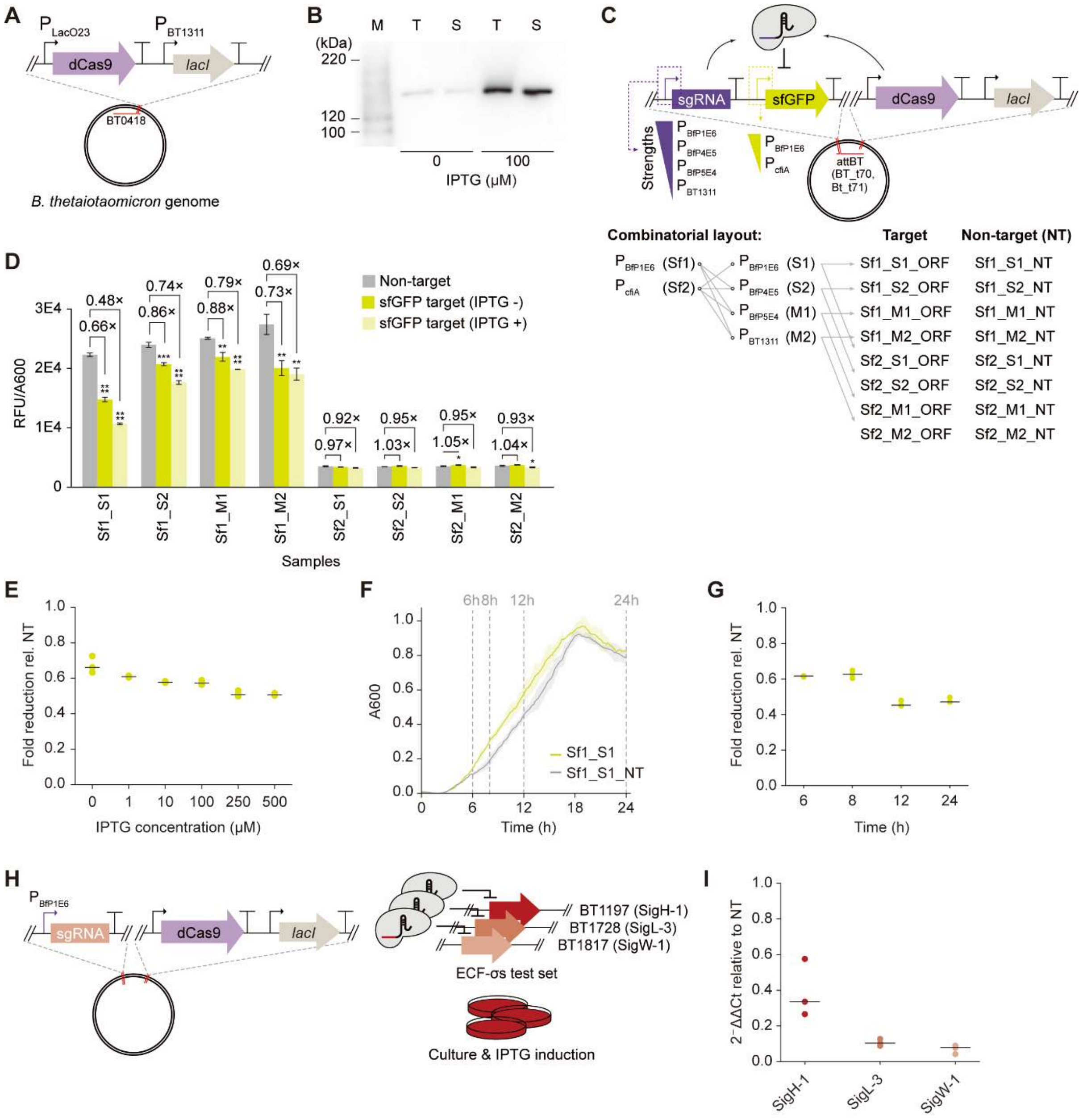

Figure S1: Implementing dCas9-based gene repression system in *B. thetaiotaomicron*. **(A)** A dCas9 expression cassette under the control of PLacO23, an IPTG-inducible promoter, was genomically integrated into the BT0418 locus. **(B)** Western blot of total cell lysates (T) and soluble fractions (S) from dCas9-expressing cells; M, molecular-weight marker (LC5602, Invitrogen). **(C)** sfGFP reporter design and combinatorial expression construct. sfGFP was expressed from four promoters of distinct strength and sgRNAs from two promoters, yielding eight ORF-targeting and eight non-targeting (NT) strains. **(D)** A_{600}-normalized sfGFP fluorescence (relative fluorescence units, RFU) in NT and ORF-targeting strains without (–) or

with (+) 100 μM IPTG. Fold changes relative to NT are indicated above each bar. Statistical significance was assessed by two-tailed Student's t-test (* *p-val* < 0.05; ** *p-val* < 0.01; *** *p-val* < 0.001; **** *p-val* < 0.0001). **(E)** Dose–response of fold reduction in RFU/A_{600} for Sf1_S1_ORF relative to Sf1_S1_NT across IPTG concentrations. **(F)** Growth curves of Sf1_S1_ORF and Sf1_S1_NT in BHIS medium with 100 μM IPTG. Vertical dashed lines mark time points used for fluorescence measurements in panel G. **(G)** Fold reduction in RFU/A600 for Sf1_S1_ORF relative to Sf1_S1_NT at 6, 8, 12 and 24h post induction. **(H)** Schematic of the ECF-σ-targeting dCas9-based repression system. PBfP1E6 drives the expression of sgRNA. Three candidate ECF-σs – BT1197 (SigH-1), BT1728 (SigL-3), and BT1817 (SigW-1) are chosen based on their expression level in **Supplementary Fig. S2A**. **(I)** qPCR quantification of transcript levels of the three target ECF-σs, expressed as $2^{-\Delta\Delta Ct}$ relative to the non-targeting (NT) control. Bars show mean of three biological replicates.

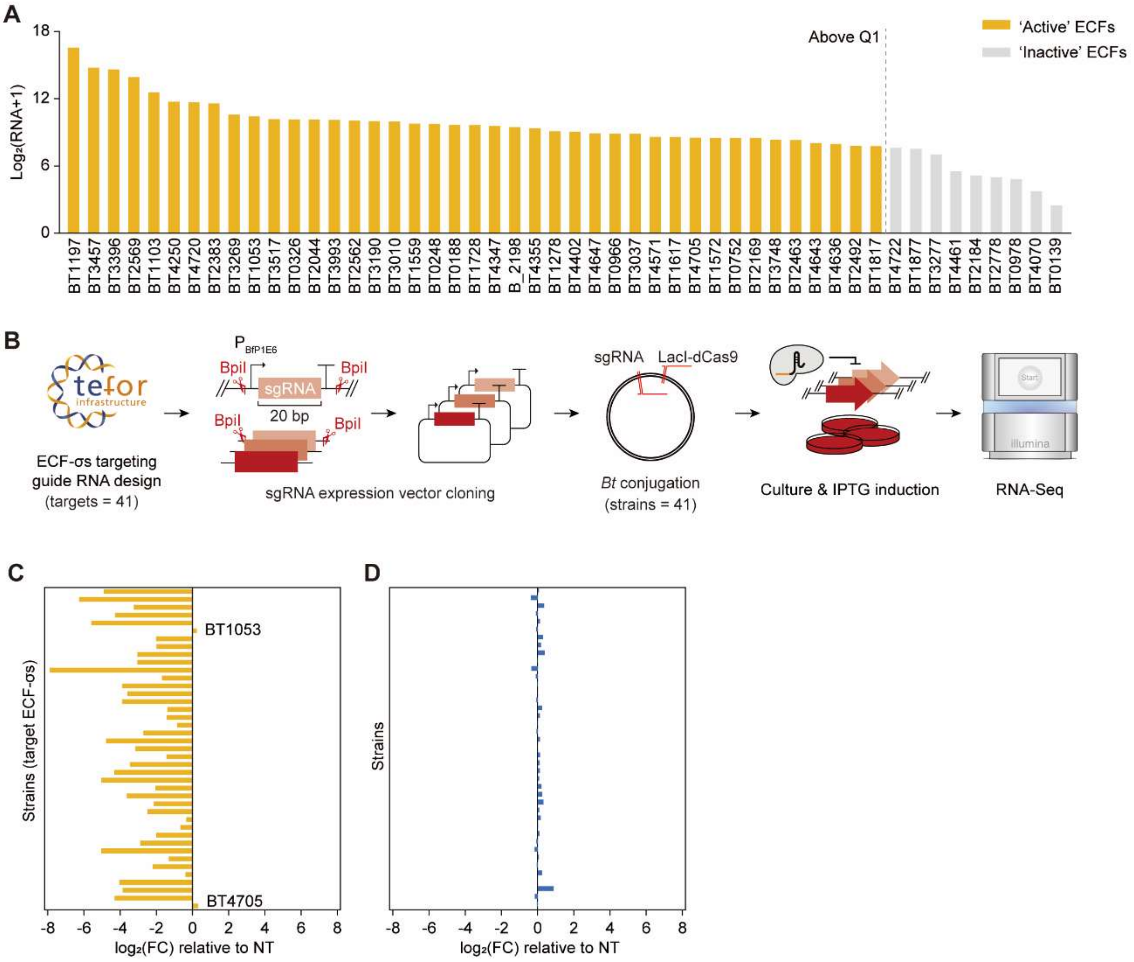

Figure S2: Selection and validation of ECF-σ repression targets. (A) Expression levels of all 50 ECF-σ factors in *B. thetaiotaomicron* during growth in CBA medium, shown as $\log_2$(RNA + 1). ECF-σ genes whose expression exceeds the first quartile (Q1) of the genome-wide distribution are highlighted in orange ('active'), whereas those below Q1 are shown in grey ('inactive'). **(B)** Workflow for ECF-σ targeting and CRISPRi library construction. Guide sequences for 41 ECF-σ genes were designed and scored using the CRISPOR platform (8). Twenty-base protospacer oligonucleotides, flanked by BpiI restriction enzyme digestion sites, were cloned into a P_BfP1E6-driven sgRNA scaffold by T4 DNA ligation. The resulting sgRNA vectors were conjugated into the dCas9-expressing *B. thetaiotaomicron* strain, cultured with IPTG to induce repression, and subjected to RNA-Seq. **(C)** $\log_2$ fold-change ($\log_2$(FC)) in transcript abundance of each targeted ECF-σ factor relative to its non-target (NT) control. Strains with positive $\log_2$(FC) of ECF-σ expression are labeled. **(D)** $\log_2$FC of the 'housekeeping' gene BT1311 in the same CRISPRi strains.

271 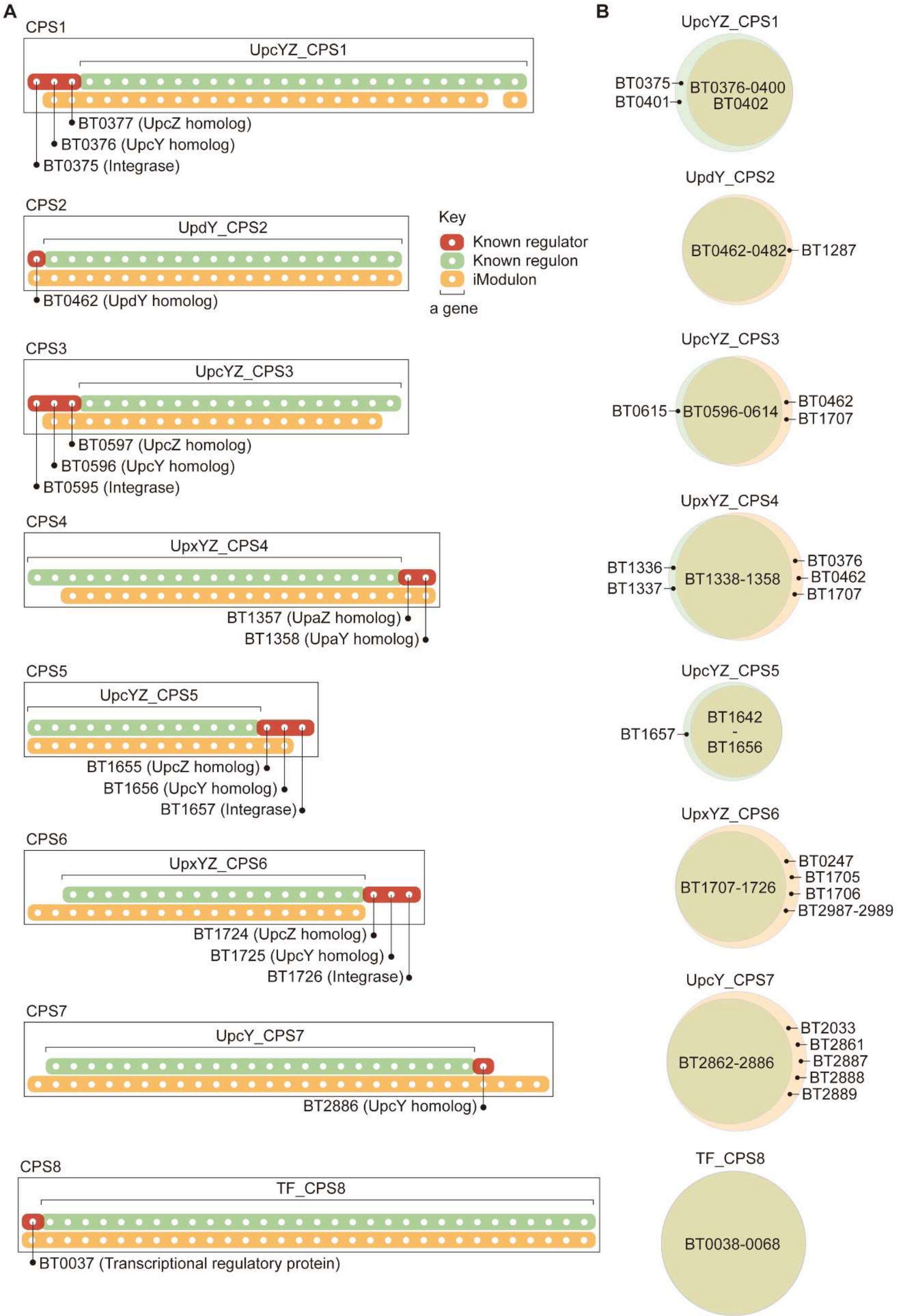
A
CPS1
UpcYZ_CPS1
BT0377 (UpcZ homolog)
BT0376 (UpcY homolog)
BT0375 (Integrase)
CPS2
UpdY_CPS2
BT0462 (UpdY homolog)
Key
Known regulator
Known regulon
iModulon
a gene
CPS3
UpcYZ_CPS3
BT0597 (UpcZ homolog)
BT0596 (UpcY homolog)
BT0595 (Integrase)
CPS4
UpxYZ_CPS4
BT1357 (UpaZ homolog)
BT1358 (UpaY homolog)
CPS5
UpcYZ_CPS5
BT1655 (UpcZ homolog)
BT1656 (UpcY homolog)
BT1657 (Integrase)
CPS6
UpxYZ_CPS6
BT1724 (UpcZ homolog)
BT1725 (UpcY homolog)
BT1726 (Integrase)
CPS7
UpcY_CPS7
BT2886 (UpcY homolog)
CPS8
TF_CPS8
BT0037 (Transcriptional regulatory protein)
B
UpcYZ_CPS1
BT0375
BT0401
BT0376-0400
BT0402
UpdY_CPS2
BT0462-0482
BT1287
UpcYZ_CPS3
BT0615
BT0596-0614
BT0462
BT1707
UpxYZ_CPS4
BT1336
BT1337
BT1338-1358
BT0376
BT0462
BT1707
UpcYZ_CPS5
BT1657
BT1642
-
BT1656
UpxYZ_CPS6
BT1707-1726
BT0247
BT1705
BT1706
BT2987-2989
UpcY_CPS7
BT2862-2886
BT2033
BT2861
BT2887
BT2888
BT2889
TF_CPS8
BT0038-0068

Figure S3: Capsular polysaccharide (CPS) biosynthetic clusters identified by ICA analysis. (A) Comparison of iModulon-predicted CPS biosynthetic cluster genes with known predictions. Each gene is represented by a dot. Light green and red strips indicate previously known/predicted CPS genes and transcription factors, respectively. Orange strips show iModulon predictions, displaying only genes immediately adjacent to known CPS clusters. **(B)** Venn diagrams illustrating all unique and overlapping genes between previously known CPS genes and those predicted by iModulon.

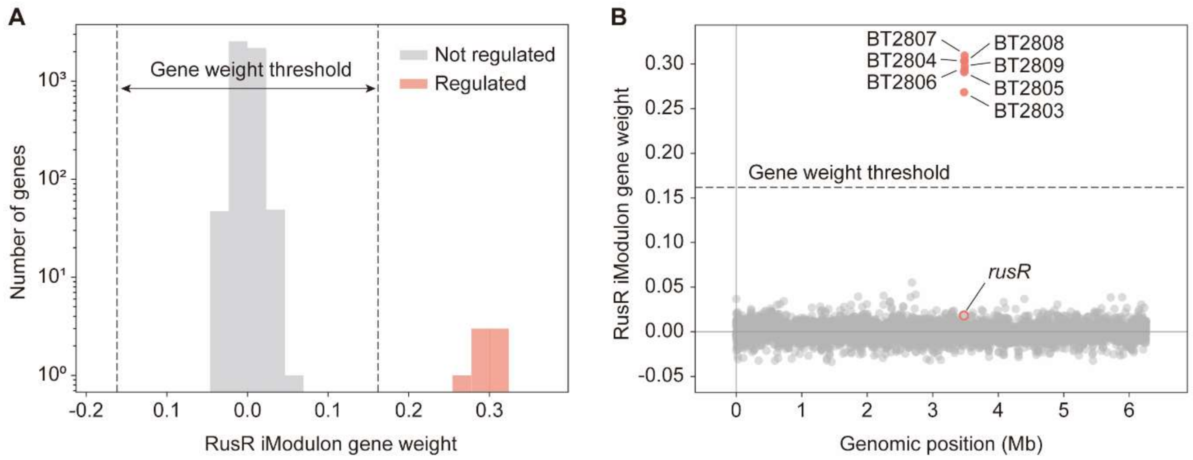

Figure S4: Genes delineated as co-regulated gene sets in the RusR iModulon. (A) RusR iModulon visualized in a histogram. Dotted vertical lines indicate gene weight threshold, beyond which gene weights significantly differ from background noise. **(B)** Gene weight plot of RusR iModulon. Genes belong to the RusR iModulon are marked in light red. *rusR* regulator, which falls below the gene weight threshold, is marked in light red border.

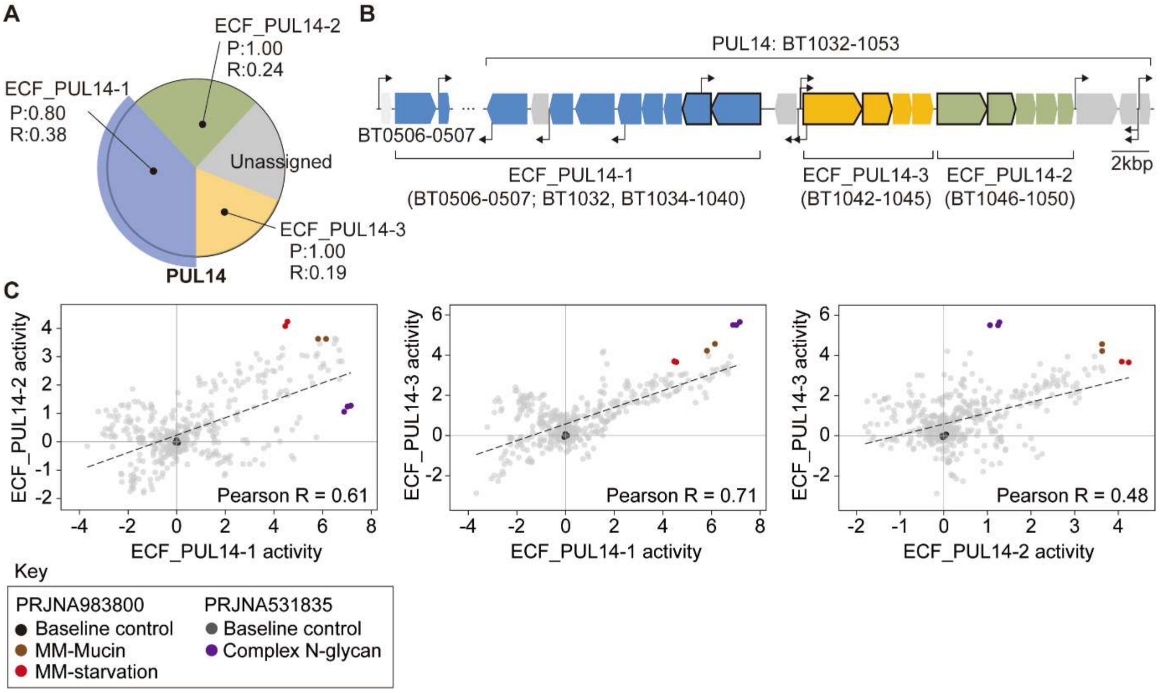

Figure S5: Three independently-regulated operons proposed by iModulon. (A) A pie chart showing the proportion, precision, and recall metrics of the three ECF_PUL14 iModulons (ECF_PUL14-1, -2, and -3). Blue, green, and orange pies represent ECF_PUL14-1, -2, and -3, respectively. SusCD homologs are outlined in black borders. **(B)** The transcription architecture of PUL14 and other genes (BT0506-0507). Transcription start sites were obtained from the Theta-Base (38). **(C)** Pairwise comparison of iModulon activity between ECF_PUL14-1, -2, and -3 across 153 different culture conditions and genetic backgrounds in the BtModulome.

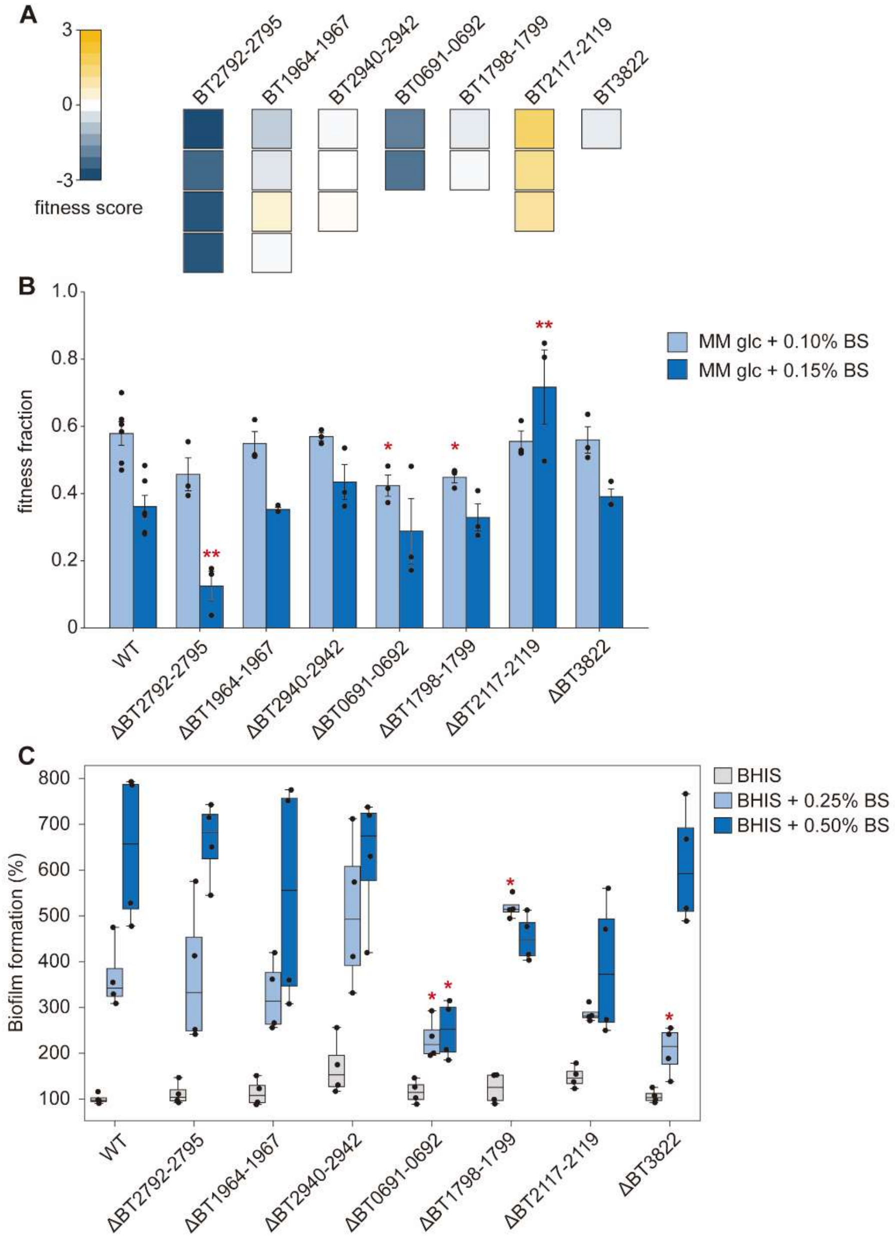

Figure S6: Bile salt-induced phenotypic changes in the knockout mutants of the members of the Bile salt efflux iModulon. (A) Fitness of the Transposon-insertion mutants exposed to 0.5 mg/mL of bile salts (in Varel Bryant medium glucose; pH7). The fitness data were retrieved from the Fitness Browser (https://fit.genomics.lbl.gov) (27). **(B)** Changes in specific growth rates of the knockout mutants in anaerobic glucose (0.5% w/v; MM-glc) with 0.10% or 0.15% bile salts, compared to MM-glc without bile salts. Mean specific growth rates of the wild-type and the mutants in MM-glc were adjusted to 1.0. **(C)** Comparison of biofilm formation capacity in response to bile salts in the wild-type and the knockout mutants. Mean of biofilm formation in wild-type was adjusted to 100%. Error bar represents standard

deviations between biological replicates. na: data not available. Statistical significance corresponds to two-sided independent t-test, where * p-val < 0.05; ** p-val < 0.01.

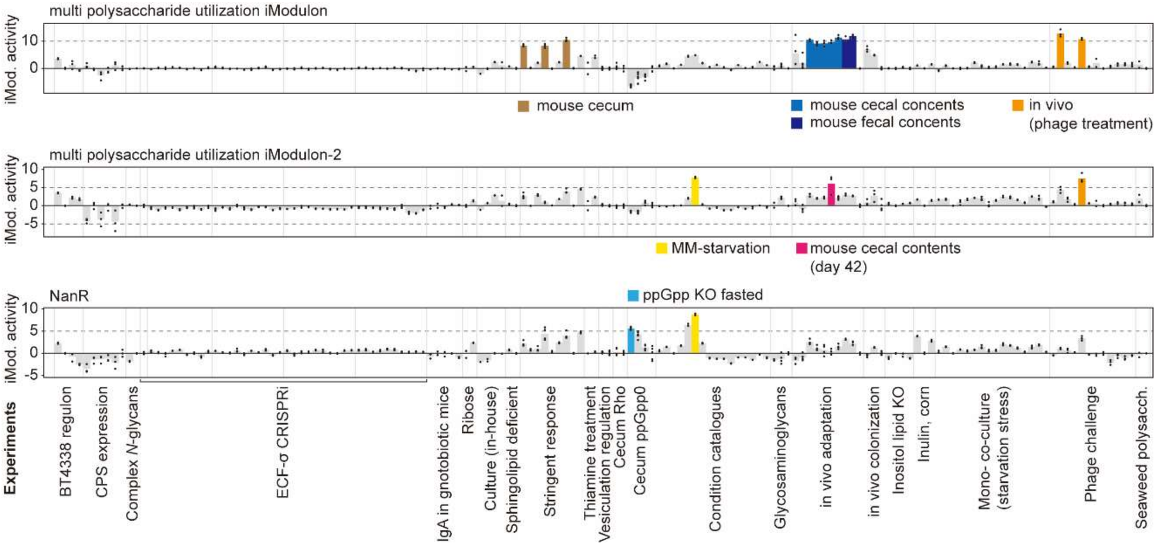

Figure S7: The activity profiles of iModulons in the 'closest match' category. iModulon activities of multi polysaccharide utilization, multi polysaccharide utilization-2, and NanR iModulon. Colored bars represent experimental conditions with mean activities above arbitrary thresholds (shown in dotted lines). Dots on each bar represent biological replicates.

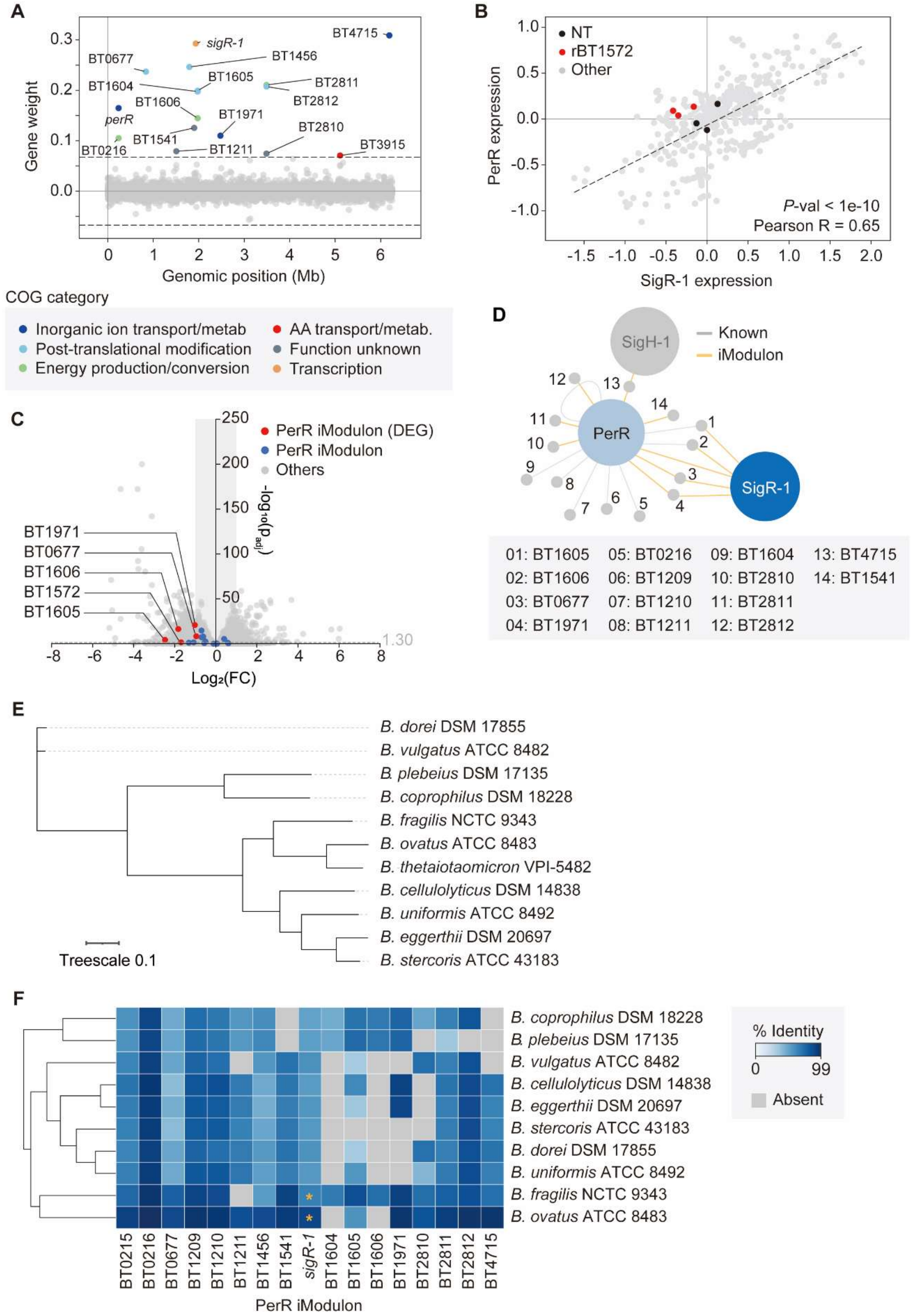

Figure S8: The PerR iModulon. (A) Gene weight in the PerR iModulon, colored circles represent membership of the iModulon. Colors denote COG categories. **(B)** Comparison of

gene expression between *perR* and *sigR-1* across all samples in BtModulome. Black dots denote CRISPRi samples with non-target sgRNA (NT), red dots are *sigR-1* targeting CRISPRi samples (rBT1572). **(C)** Differentially expressed genes (DEGs) analysis of the PerR iModulon membership in rBT1572 using NT as the control. Genes with statistically significant changes in gene expression (Padj < 0.05) were marked as DEGs. Interestingly, BT1209 and BT1210, the known regulons of PerR that are absent in the PerR iModulon (Fig. 2B) were also differentially expressed in the *sigR-1* repressed strain. The exclusion of these two may be due to highly variable transcription regulation patterns, or weak TF binding. **(D)** Proposed regulatory association in the PerR iModulon based on the DEG analysis. **(E)** Phylogenetic tree constructed using the eleven *Bacteroides* type strain proteomes. **(F)** Heatmap showing clustered percentage identity (metric: Euclidean, method: weighted) of each *B. thetaiotaomicron* PerR iModulon against its orthologous in the ten other *Bacteroides* proteomes. BLASTp hits were filtered using an e-value cutoff of 1e-20 and a minimum sequence identity of 30%. Orthologs of SigR-1 marked with an orange asterisk belong to the ECF-σ subgroup of 114s7.

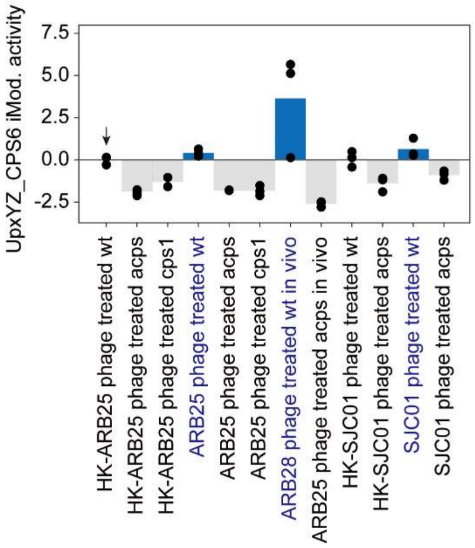

Figure S9: An activity plot of the UpxYZ_CPS6 iModulon in 'phage challenge' project. Bars with positive coefficient of iModulon activity are highlighted in blue. The arrow indicates the baseline control sample. CPS6-expressing *B. thetaiotaomicron* showed reduced sensitivity toward ARB25 and SJC01 phage in liquid culture environment, which likely explains the increased UpxYZ_CPS6 iModulon activity in "ARB25 phage treated wt" and "SJC01 phage treated wt" experiments (14). However, the CPS6 strain was permissive to ARB25 infection *in vivo* and plate culture; accordingly, the increased UpxYZ_CPS6 iModulon activity is attributed to increase *cps6* expression *in vivo* (13,14).

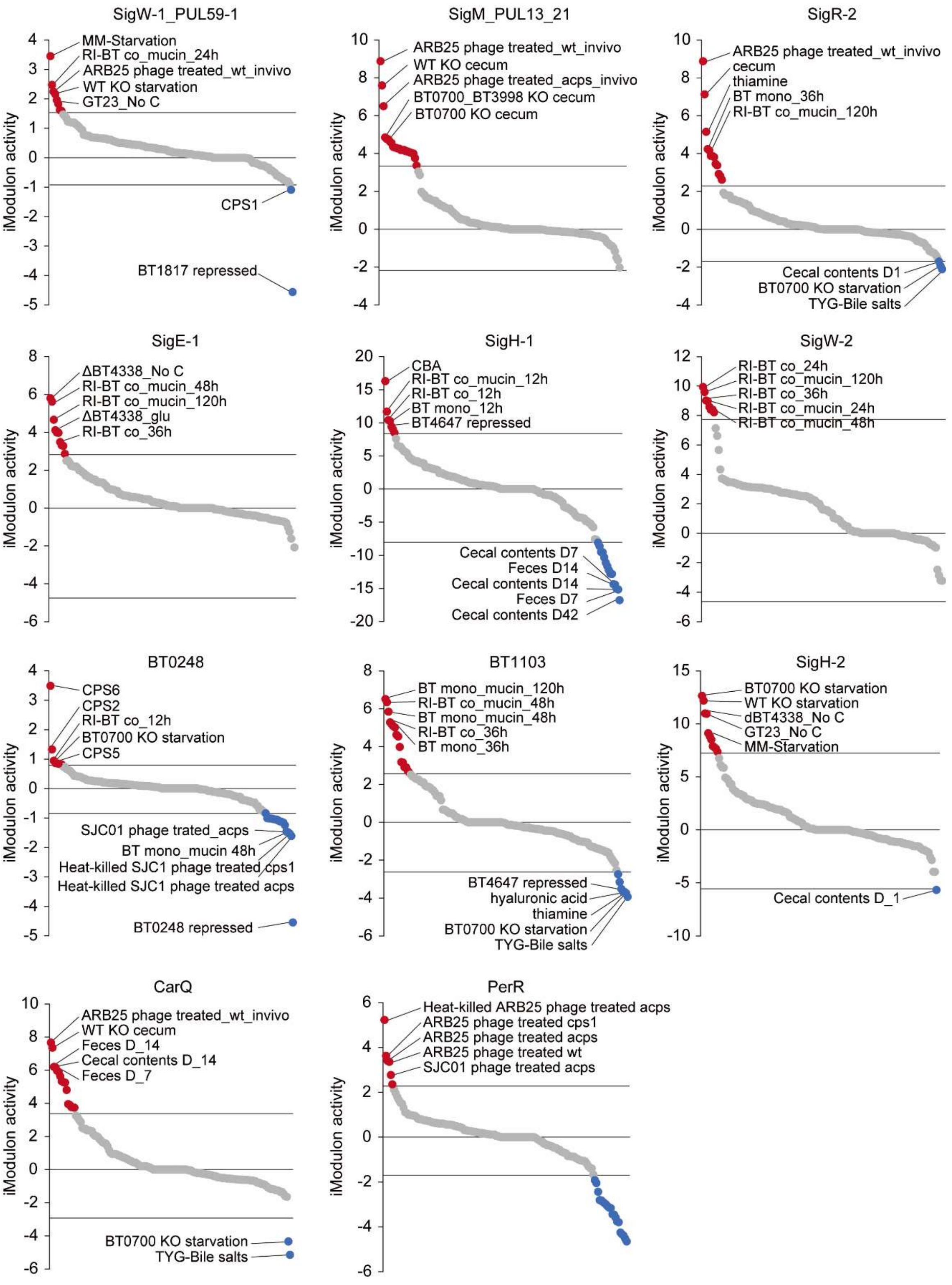

Figure S10: Condition-specific activities of the iModulons associated with ECF-σs. Experimental conditions with iModulon activity above the third quartile (Q3) and below the first quartile (Q1) are highlighted in red and blue, respectively.

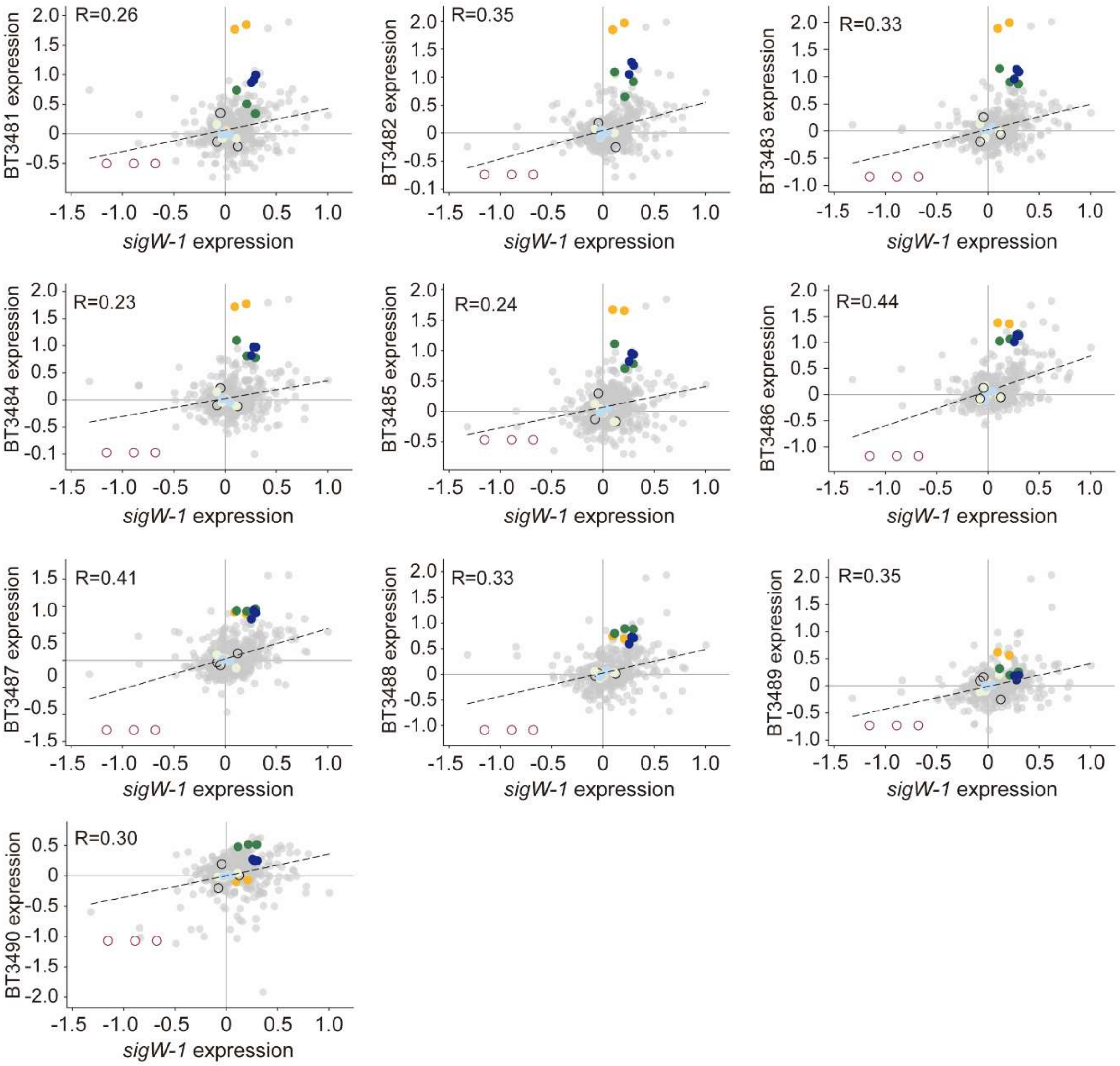

Figure S11: Comparison of gene expression between *sigW-1* and BT3481-BT3490 operon. Colors correspond to those in Fig. 4E.

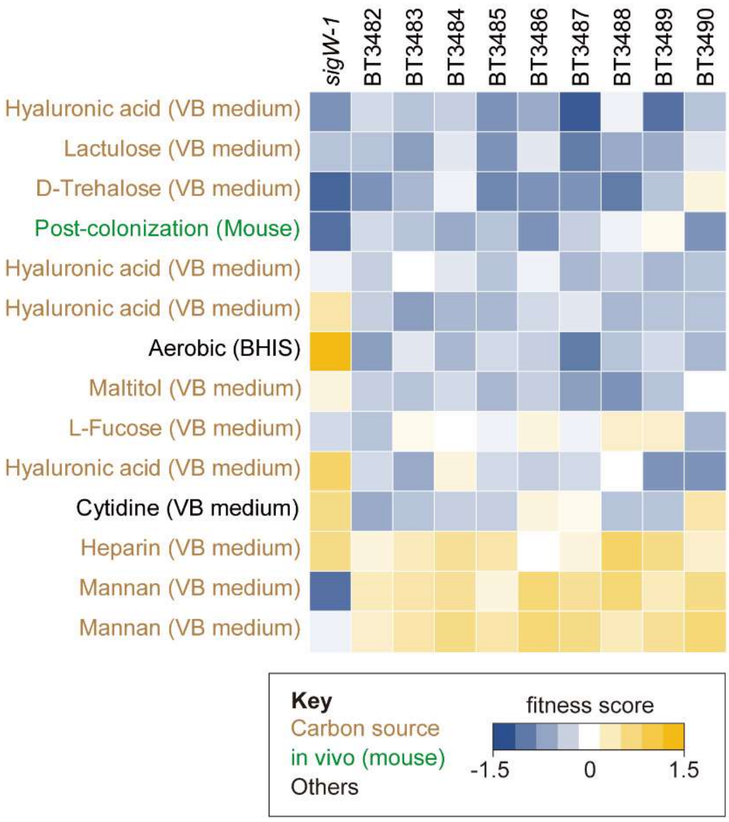

Figure S12: Fitness profiles of knockout (Tn-Seq) derivatives of s*igW-1* and its proposed regulons. Heatmap depicting gene fitness across various conditions, including different carbon sources and *in vivo* mouse experiments. Data were obtained from the Fitness Brower (https://fit.genomics.lbl.gov) (3). Fitness data for BT3481 were omitted, and conditions categorized as 'stress' in the Fitness Browser database were excluded from the analysis.

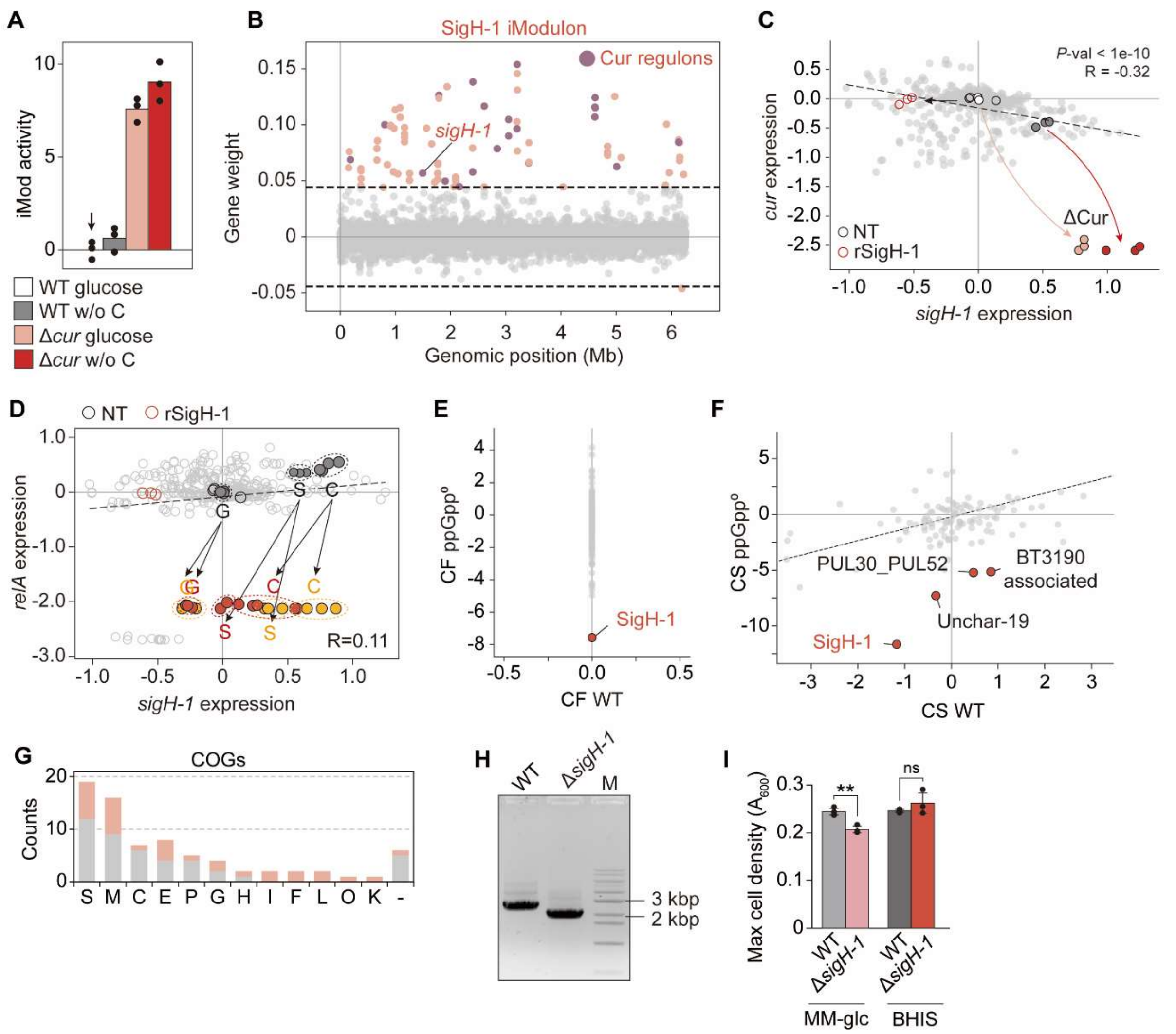

Figure S13: Extended figure for the SigH-1 iModulon. (A) iModulon activity under induced starvation in wild-type and Δ*cur* background in 'BT4338 regulon' project. **(B)** Scatter plot showing the gene weights within the iModulon. Cur regulons are shaded in dark pink. **(C)** Gene expression profiles of *sigH-1* and *cur*. Color schemes are identical to those in panel A. Grey dots are the rest of the BtModulme. **(D)** Gene expression profiles of *sigH*-1 and *relA* (BT0700) in the BtModulme. **(E-F)** Differential iModulon activity graph between **(E)** wild-type fed *in vivo* (CF WT), and ppGpp^0 mutant fed *in vivo* (CF, ppGpp^0), and **(F)** between CF WT and ppGpp^0 mutant fasted *in vivo* (CS ppGpp^0). **(G)** COG category of genes in the SigH-1 iModulon and the proposed *sigH-1* regulons. Color code is identical to Fig. 5E. COG abbreviations – S: unknown, M: cell wall/membrane/envelope biogenesis, C: energy production and conversion, E: amino acid transport and metabolism, P: inorganic ion transport and metabolism, G: carbohydrate transport and metabolism, H: coenzyme

transport and metabolism, I: lipid transport and metabolism, F: nucleotide transport and metabolism, L: replication, recombination and repair, O: post-translational modification, K: transcription, -: no COG assignment. **(H)** Gel electrophoresis of PCR amplicons from the *sigH-1* genomic region in *B. thetaiotaomicron* wild-type and Δ*sigH-1* strains. **(I)** Maximum cell density of the wild-type and Δ*sigH-1* in each medium.

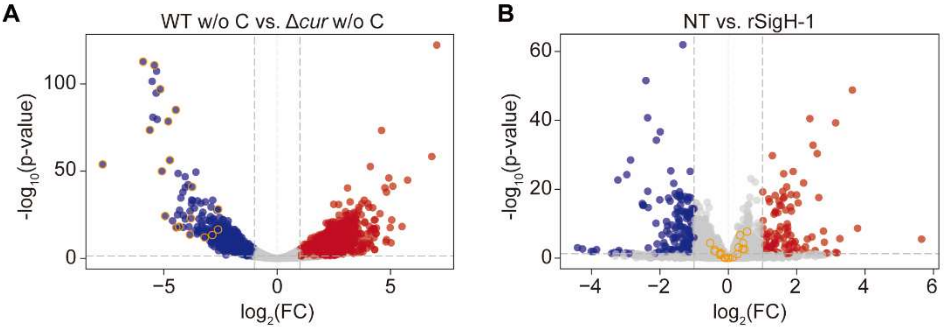

Figure S14: Comparison of fold-change gene expression profiles of key Cur regulons between Δ*cur* under starvation and rSigH-1. (A-B) Volcano plot of the $\log_2$FC versus the -$\log_{10}$ of the corresponding *p*-val for genes in two dataset – **(A)** between WT and Δ*cur* exposed to carbon limitation (24); **(B)** between NT control and rSigH-1. The Cur regulons that are associated with carbohydrate utilization and gut colonization – *fusA2*, BT0350, BT0355, *araM*, BT0792, BT0793, BT1432, BT1433, BT1434, BT0791, BT0617, BT0618, BT1277, BT4296, BT4295, BT4298, BT2818, BT2819, BT1450 – identified by Townsend et al. (24) are marked in orange.

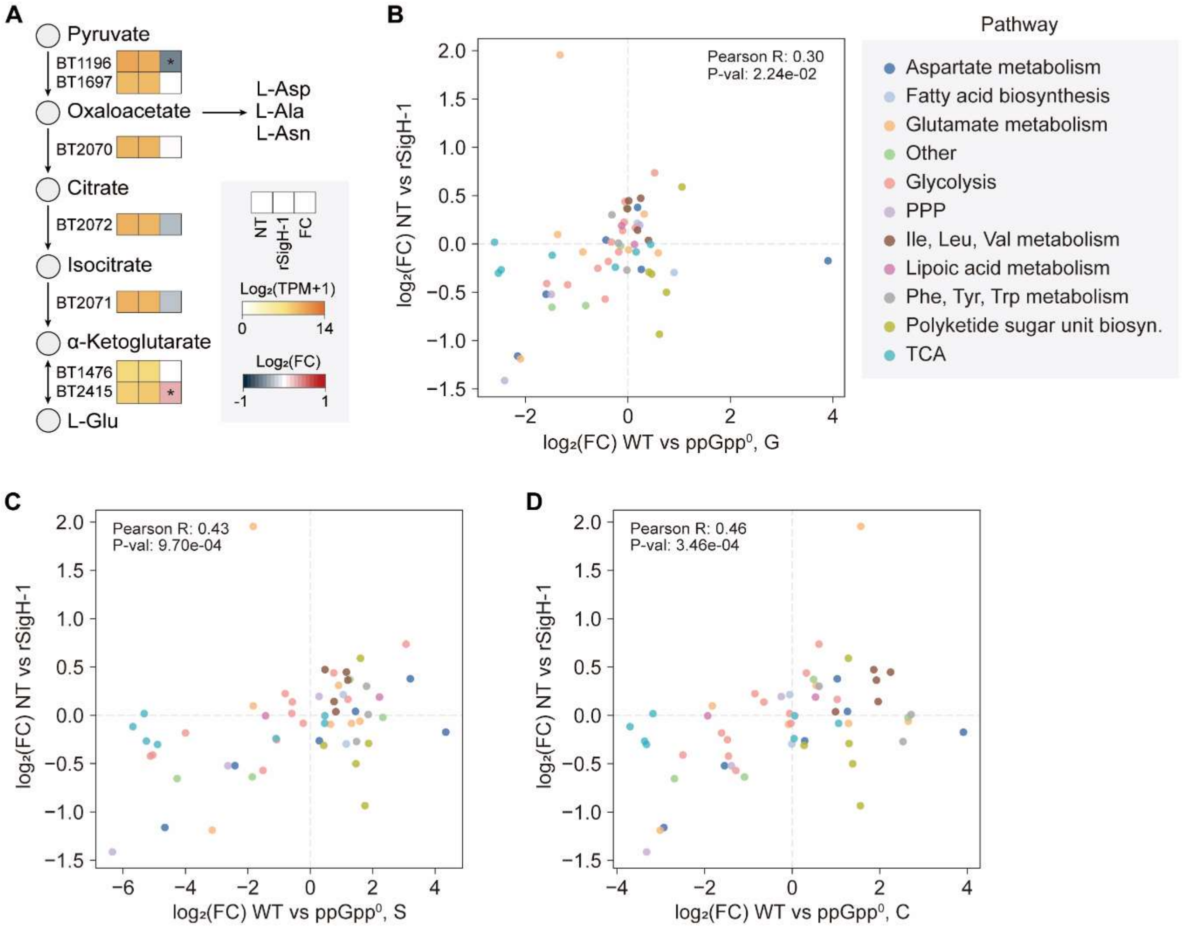

Figure S15: DEG profile analysis of genes significantly altered in the ppGpp^0 background. **(A)** Expression changes in the α-ketoglutarate biosynthetic module in rSigH-1 compared to NT control. Asterisk represents fold-changes with p_{adj} < 0.01. **(B-D)** Scatter plot of DEG profiles between rSigH-1 and **(B)** ppGpp^0 under glucose, **(C)** ppGpp^0 under starvation, and **(D)** ppGpp^0 *in vivo* glucose. Genes are based on Table S4C of Schofield et al. (9).

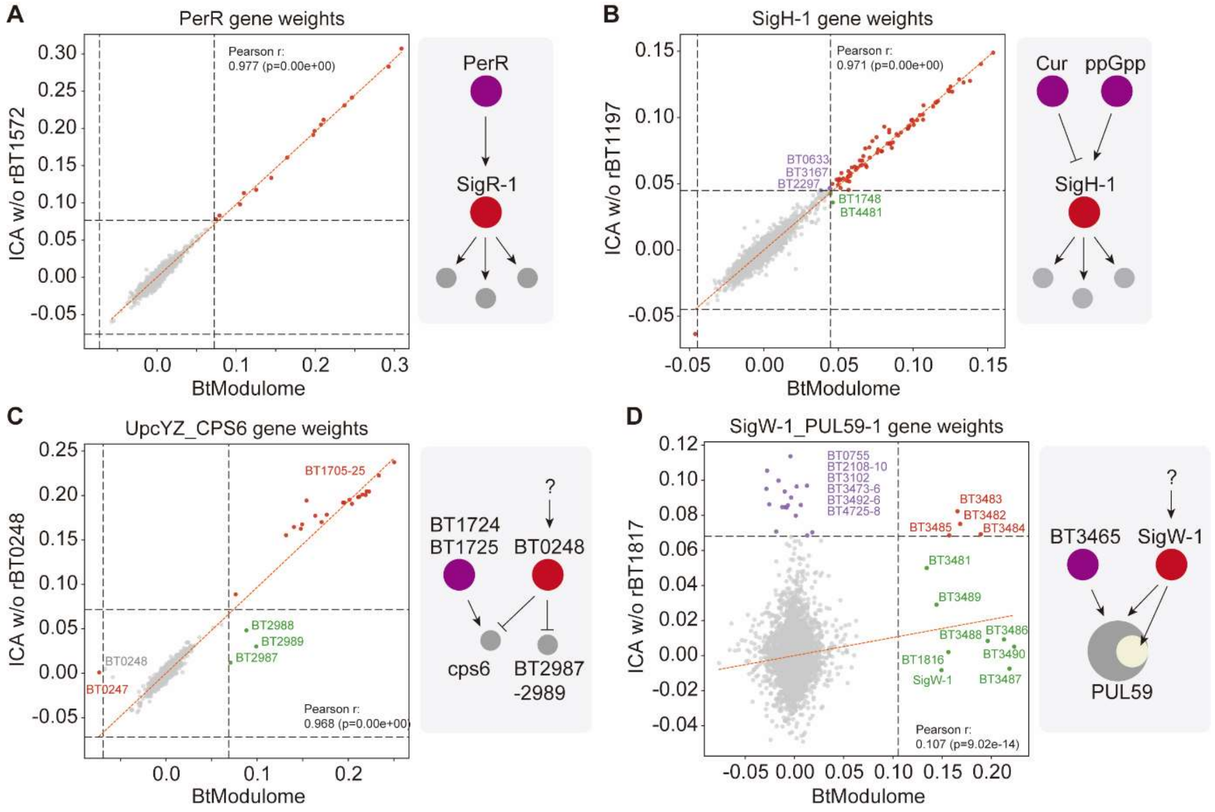

Figure S16: Sensitivity of iModulon reconstruction to removal of ECF-σ repression datasets. Scatter plots of gene weights between the BtModulome (x-axis) with ICA re-run after removing all RNA-seq samples corresponding to CRISPRi repression of individual ECF-σ factors (y-axis). Each dot represents a gene; significantly weighted members of the respective iModulon are colored by functional grouping. Red: genes shared between two iModulons; green: genes unique to iModulon in the BtModulome; purple: genes unique to iModulon in the ICA re-runs. Simplified network schematics (right) summarize the regulatory context inferred for each case. (**A**) Removal of SigR-1 (BT1572) repression data shows minimal effect (r = 0.977), as SigR-1 is embedded within the PerR oxidative stress regulon. (**B**) Removal of SigH-1 (BT1197) data also shows little effect (r = 0.971), consistent with hierarchical regulation by Cur and (p)ppGpp. (**C**) Removal of BT0248 data (r = 0.968) abolishes the contra-regulatory relationship between BT0248 and the cps6 cluster (BT1705–25) and lipoproteins (BT2987–2989). (**D**) Removal of SigW-1 (BT1817) data (r = 0.107) results in collapse of the PUL59-associated iModulon, indicating this regulon is uniquely resolved by direct perturbation.

REFERENCE

1. Mimee, M., Tucker, A.C., Voigt, C.A. and Lu, T.K. (2015) Programming a human commensal bacterium, *Bacteroides thetaiotaomicron*, to sense and respond to stimuli in the murine gut microbiota. *Cell Syst*, **1**, 62-71.
2. Prezza, G., Liao, C., Reichardt, S., Beisel, C.L. and Westermann, A.J. (2024) CRISPR-based screening of small RNA modulators of bile susceptibility in *Bacteroides thetaiotaomicron*. *Proc Natl Acad Sci U S A*, **121**, e2311323121.
3. Prezza, G. and Westermann, A.J. (2024) CRISPR interference-based functional small RNA genomics. *Methods Mol Biol*, **2741**, 101-116.
4. Kang, M., Kim, K. and Cho, B.K. (2024) CRISPRi-driven genetic screening for designing novel microbial phenotypes. *Methods Mol Biol*, **2760**, 117-132.
5. Pedelacq, J.D., Cabantous, S., Tran, T., Terwilliger, T.C. and Waldo, G.S. (2006) Engineering and characterization of a superfolder green fluorescent protein. *Nat Biotechnol*, **24**, 79-88.
6. Whitaker, W.R., Shepherd, E.S. and Sonnenburg, J.L. (2017) Tunable expression tools enable single-cell strain distinction in the gut microbiome. *Cell*, **169**, 538-546 e512.
7. Xu, J., Bjursell, M.K., Himrod, J., Deng, S., Carmichael, L.K., Chiang, H.C., Hooper, L.V. and Gordon, J.I. (2003) A genomic view of the human-*Bacteroides thetaiotaomicron* symbiosis. *Science*, **299**, 2074-2076.
8. Concordet, J.P. and Haeussler, M. (2018) CRISPOR: intuitive guide selection for CRISPR/Cas9 genome editing experiments and screens. *Nucleic Acids Res*, **46**, W242-W245.
9. Schofield, W.B., Zimmermann-Kogadeeva, M., Zimmermann, M., Barry, N.A. and Goodman, A.L. (2018) The stringent response determines the ability of a commensal bacterium to survive starvation and to persist in the gut. *Cell Host Microbe*, **24**, 120-132 e126.
10. Kostopoulos, I., Aalvink, S., Kovatcheva-Datchary, P., Nijsse, B., Backhed, F., Knol, J., de Vos, W.M. and Belzer, C. (2021) A continuous battle for host-derived glycans between a mucus specialist and a glycan generalist *in vitro* and *in vivo*. *Front Microbiol*, **12**, 632454.
11. Liu, B., Garza, D.R., Gonze, D., Krzynowek, A., Simoens, K., Bernaerts, K., Geirnaert, A. and Faust, K. (2023) Starvation responses impact interaction dynamics of human gut bacteria *Bacteroides thetaiotaomicron* and *Roseburia intestinalis*. *ISME J*, **17**, 1940-1952.
12. Kennedy, M.S., Zhang, M., DeLeon, O., Bissell, J., Trigodet, F., Lolans, K., Temelkova, S., Carroll, K.T., Fiebig, A., Deutschbauer, A. *et al.* (2023) Dynamic genetic adaptation of *Bacteroides thetaiotaomicron* during murine gut colonization. *Cell Rep*, **42**, 113009.
13. Porter, N.T., Canales, P., Peterson, D.A. and Martens, E.C. (2017) A subset of polysaccharide capsules in the human symbiont *Bacteroides thetaiotaomicron* promote increased competitive fitness in the mouse gut. *Cell Host Microbe*, **22**, 494-506 e498.
14. Porter, N.T., Hryckowian, A.J., Merrill, B.D., Fuentes, J.J., Gardner, J.O., Glowacki, R.W.P., Singh, S., Crawford, R.D., Snitkin, E.S., Sonnenburg, J.L. *et al.* (2020) Phase-variable capsular polysaccharides and lipoproteins modify bacteriophage susceptibility in *Bacteroides thetaiotaomicron*. *Nat Microbiol*, **5**, 1170-1181.

15. Krypotou, E., Townsend, G.E., Gao, X., Tachiyama, S., Liu, J., Pokorzynski, N.D., Goodman, A.L. and Groisman, E.A. (2023) Bacteria require phase separation for fitness in the mammalian gut. *Science*, **379**, 1149-1156.
16. Ryan, D., Bornet, E., Prezza, G., Alampalli, S.V., Franco de Carvalho, T., Felchle, H., Ebbecke, T., Hayward, R.J., Deutschbauer, A.M., Barquist, L. *et al.* (2024) An expanded transcriptome atlas for *Bacteroides thetaiotaomicron* reveals a small RNA that modulates tetracycline sensitivity. *Nat Microbiol*, **9**, 1130-1144.
17. Overbeeke, A., Hausmann, B., Nikolov, G., Pereira, F.C., Herbold, C.W. and Berry, D. (2022) Nutrient niche specificity for glycosaminoglycans is reflected in polysaccharide utilization locus architecture of gut *Bacteroides* species. *Front Microbiol*, **13**, 1033355.
18. Bedu-Ferrari, C., Biscarrat, P., Pepke, F., Vati, S., Chaudemanche, C., Castelli, F., Chollet, C., Rue, O., Hennequet-Antier, C., Langella, P. *et al.* (2024) In-depth characterization of a selection of gut commensal bacteria reveals their functional capacities to metabolize dietary carbohydrates with prebiotic potential. *mSystems*, **9**, e0140123.
19. Briliute, J., Urbanowicz, P.A., Luis, A.S., Basle, A., Paterson, N., Rebello, O., Hendel, J., Ndeh, D.A., Lowe, E.C., Martens, E.C. *et al.* (2019) Complex N-glycan breakdown by gut *Bacteroides* involves an extensive enzymatic apparatus encoded by multiple co-regulated genetic loci. *Nat Microbiol*, **4**, 1571-1581.
20. Glowacki, R.W.P., Pudlo, N.A., Tuncil, Y., Luis, A.S., Sajjakulnukit, P., Terekhov, A.I., Lyssiotis, C.A., Hamaker, B.R. and Martens, E.C. (2020) A ribose-scavenging system confers colonization fitness on the human gut symbiont *Bacteroides thetaiotaomicron* in a diet-specific manner. *Cell Host Microbe*, **27**, 79-92 e79.
21. Pudlo, N.A., Pereira, G.V., Parnami, J., Cid, M., Markert, S., Tingley, J.P., Unfried, F., Ali, A., Varghese, N.J., Kim, K.S. *et al.* (2022) Diverse events have transferred genes for edible seaweed digestion from marine to human gut bacteria. *Cell Host Microbe*, **30**, 314-328 e311.
22. Ontai-Brenning, A., Hamchand, R., Crawford, J.M. and Goodman, A.L. (2023) Gut microbes modulate (p)ppGpp during a time-restricted feeding regimen. *mBio*, **14**, e0190723.
23. Pardue, E.J., Sartorio, M.G., Jana, B., Scott, N.E., Beatty, W.L., Ortiz-Marquez, J.C., Van Opijnen, T., Hsu, F.F., Potter, R.F. and Feldman, M.F. (2024) Dual membrane-spanning anti-sigma factors regulate vesiculation in *Bacteroides thetaiotaomicron*. *Proc Natl Acad Sci U S A*, **121**, e2321910121.
24. Townsend, G.E., 2nd, Han, W., Schwalm, N.D., 3rd, Hong, X., Bencivenga-Barry, N.A., Goodman, A.L. and Groisman, E.A. (2020) A master regulator of *Bacteroides thetaiotaomicron* gut colonization controls carbohydrate utilization and an alternative protein synthesis factor. *mBio*, **11**.
25. Martens, E.C., Chiang, H.C. and Gordon, J.I. (2008) Mucosal glycan foraging enhances fitness and transmission of a saccharolytic human gut bacterial symbiont. *Cell Host Microbe*, **4**, 447-457.
26. Shipman, J.A., Berleman, J.E. and Salyers, A.A. (2000) Characterization of four outer membrane proteins involved in binding starch to the cell surface of *Bacteroides thetaiotaomicron*. *J Bacteriol*, **182**, 5365-5372.
27. Liu, H., Shiver, A.L., Price, M.N., Carlson, H.K., Trotter, V.V., Chen, Y., Escalante, V., Ray, J., Hern, K.E., Petzold, C.J. *et al.* (2021) Functional genetics of human gut commensal

Bacteroides thetaiotaomicron reveals metabolic requirements for growth across environments. *Cell Rep*, **34**, 108789.

28. Bechon, N., Mihajlovic, J., Lopes, A.A., Vendrell-Fernandez, S., Deschamps, J., Briandet, R., Sismeiro, O., Martin-Verstraete, I., Dupuy, B. and Ghigo, J.M. (2022) *Bacteroides thetaiotaomicron* uses a widespread extracellular DNase to promote bile-dependent biofilm formation. *Proc Natl Acad Sci U S A*, **119**.
29. Bechon, N., Mihajlovic, J., Vendrell-Fernandez, S., Chain, F., Langella, P., Beloin, C. and Ghigo, J.M. (2020) Capsular Polysaccharide Cross-Regulation Modulates Bacteroides thetaiotaomicron Biofilm Formation. *mBio*, **11**.
30. Mihajlovic, J., Bechon, N., Ivanova, C., Chain, F., Almeida, A., Langella, P., Beloin, C. and Ghigo, J.M. (2019) A Putative Type V Pilus Contributes to *Bacteroides thetaiotaomicron* Biofilm Formation Capacity. *J Bacteriol*, **201**.
31. Lopes, A.A., Vendrell-Fernandez, S., Deschamps, J., Georgeault, S., Cokelaer, T., Briandet, R. and Ghigo, J.M. (2024) Bile-induced biofilm formation in *Bacteroides thetaiotaomicron* requires magnesium efflux by an RND pump. *mBio*, **15**, e0348823.
32. Rychel, K., Sastry, A.V. and Palsson, B.O. (2020) Machine learning uncovers independently regulated modules in the *Bacillus subtilis* transcriptome. *Nat Commun*, **11**, 6338.
33. Cartmell, A., Munoz-Munoz, J., Briggs, J.A., Ndeh, D.A., Lowe, E.C., Basle, A., Terrapon, N., Stott, K., Heunis, T., Gray, J. *et al.* (2018) A surface endogalactanase in *Bacteroides thetaiotaomicron* confers keystone status for arabinogalactan degradation. *Nat Microbiol*, **3**, 1314-1326.
34. Martens, E.C., Lowe, E.C., Chiang, H., Pudlo, N.A., Wu, M., McNulty, N.P., Abbott, D.W., Henrissat, B., Gilbert, H.J., Bolam, D.N. *et al.* (2011) Recognition and degradation of plant cell wall polysaccharides by two human gut symbionts. *PLoS Biol*, **9**, e1001221.
35. Sastry, A.V., Gao, Y., Szubin, R., Hefner, Y., Xu, S., Kim, D., Choudhary, K.S., Yang, L., King, Z.A. and Palsson, B.O. (2019) The *Escherichia coli* transcriptome mostly consists of independently regulated modules. *Nat Commun*, **10**, 5536.
36. Patel, A., McGrosso, D., Hefner, Y., Campeau, A., Sastry, A.V., Maurya, S., Rychel, K., Gonzalez, D.J. and Palsson, B.O. (2024) Proteome allocation is linked to transcriptional regulation through a modularized transcriptome. *Nat Commun*, **15**, 5234.
37. Rychel, K., Decker, K., Sastry, A.V., Phaneuf, P.V., Poudel, S. and Palsson, B.O. (2021) iModulonDB: a knowledgebase of microbial transcriptional regulation derived from machine learning. *Nucleic Acids Res*, **49**, D112-D120.
38. Ryan, D., Jenniches, L., Reichardt, S., Barquist, L. and Westermann, A.J. (2020) A high-resolution transcriptome map identifies small RNA regulation of metabolism in the gut microbe *Bacteroides thetaiotaomicron*. *Nat Commun*, **11**, 3557.
